# Shorebirds wintering in Southeast Asia demonstrate trans-Himalayan flights

**DOI:** 10.1038/s41598-020-77897-z

**Published:** 2020-12-11

**Authors:** David Li, Geoffrey Davison, Simeon Lisovski, Phil F. Battley, Zhijun Ma, Shufen Yang, Choon Beng How, Doug Watkins, Philip Round, Alex Yee, Vupasana Srinivasan, Clarice Teo, Robert Teo, Adrian Loo, Chee Chiew Leong, Kenneth Er

**Affiliations:** 1 National Parks Board, Singapore, 718925 Singapore; 2 Alfred-Wegener-Institute Helmholtz Centre for Marine and Polar Research, Potsdam, Germany; 3 Wildlife & Ecology Group, Massey University, Palmerston North, 4442 New Zealand; 4 Ministry of Education Key Laboratory for Biodiversity Science and Ecological Engineering, Coastal Ecosystems Research Station of the Yangtze River Estuary, Institute of Biodiversity Science, School of Life Sciences, Fudan University, Shanghai, 200433 China; 5 Australasian Wader Studies Group, Carlton, VIC 3053 Australia; 6 Department of Biology, Mahidol University, Bangkok, 10400 Thailand

**Keywords:** Ecology, Zoology

## Abstract

Many birds wintering in the Indian subcontinent fly across the Himalayas during migration, including Bar-headed Geese (*Anser indicus*), Demoiselle Cranes (*Anthropoides virgo*) and Ruddy Shelducks (*Tadorna ferruginea*). However, little is known about whether shorebirds migrate across the Himalayas from wintering grounds beyond the Indian subcontinent. Using geolocators and satellite tracking devices, we demonstrate for the first time that Common Redshanks (*Tringa totanus*) and Whimbrels (*Numenius phaeopus*) wintering in Singapore can directly fly over the Himalayas to reach breeding grounds in the Qinghai-Tibet Plateau and north-central Russia respectively. The results also show that migratory shorebirds wintering in Southeast Asia can use both the Central Asian Flyway and the East Asian-Australasian Flyway. For Redshanks, westerly-breeding birds crossed the Himalayas while more easterly breeders on the Plateau migrated east of the Himalayas. For Whimbrels, an individual that crossed the Himalayas was probably from a breeding population that was different from the others that migrated along the coast up the East Asian-Australasian Flyway. The minimum required altitude of routes of trans-Himalayan Redshanks were no higher on average than those of eastern migrants, but geolocator temperature data indicate that birds departing Singapore flew at high elevations even when not required to by topography, suggesting that the Himalayan mountain range may be less of a barrier than assumed.

## Introduction

Many migratory birds face a variety of physical barriers such as mountains, oceans, ice cover and deserts during migration, and must either cross them or circumvent them^1–4^. Birds crossing such barriers may face physiologically harsh conditions such as extreme temperatures and humidity, or greatly reduced oxygen levels, and these routes can be energetically demanding and lack refuelling opportunities^1^. Crossing barriers may be aided by factors such as wind support, and result in a shorter migration distance and time, and be more energy efficient overall^1,5–8^. Detours to avoid barriers can reduce risks and although migration distances are typically longer^3^_,_ birds may also benefit from favourable winds, and be able to refuel during stopovers and not need to store heavy fuel reserves to the same extent^1,3,4^. One such barrier is the Himalayan mountain range, which extends 2400 km in an east–west direction in southern Asia. This separates the non-breeding grounds of migratory birds from Pakistan to southeast Asia from their breeding grounds in the Qinghai-Tibet Plateau and further north, with a barrier of mountains 5000–8000 m high. Early observations suggested that many bird species migrate through the Himalayas^9,10^ and ringing recoveries have linked populations of numerous species on either side of the range^11–13^. While trans-Himalayan movements of large waterbirds (ducks, geese and cranes) have been documented by satellite tracking^14–18^, the routes taken by smaller birds such as shorebirds are poorly known.

Ringing recovery information indicates that a large number of shorebird species that migrate along the Central Asian Flyway (CAF) between Siberia and India may cross the Himalayas^12,19–21^. Potentially up to 1.4 million shorebirds of at least 47 species wintering in the Indian subcontinent cross the Himalayas to breed on the Qinghai-Tibet Plateau or areas to the north^22^. However, it is unclear whether shorebirds from wintering grounds east of the Indian subcontinent also cross the Himalayas on migration, or take an alternative route through Yunnan, China, to avoid (or partially avoid) the Himalayas. This route might allow birds to adapt to the high-altitude environment gradually by using valleys along major rivers of this region, or they could disperse to breeding grounds at lower elevations north and east of the Plateau.

**Figure 1:**
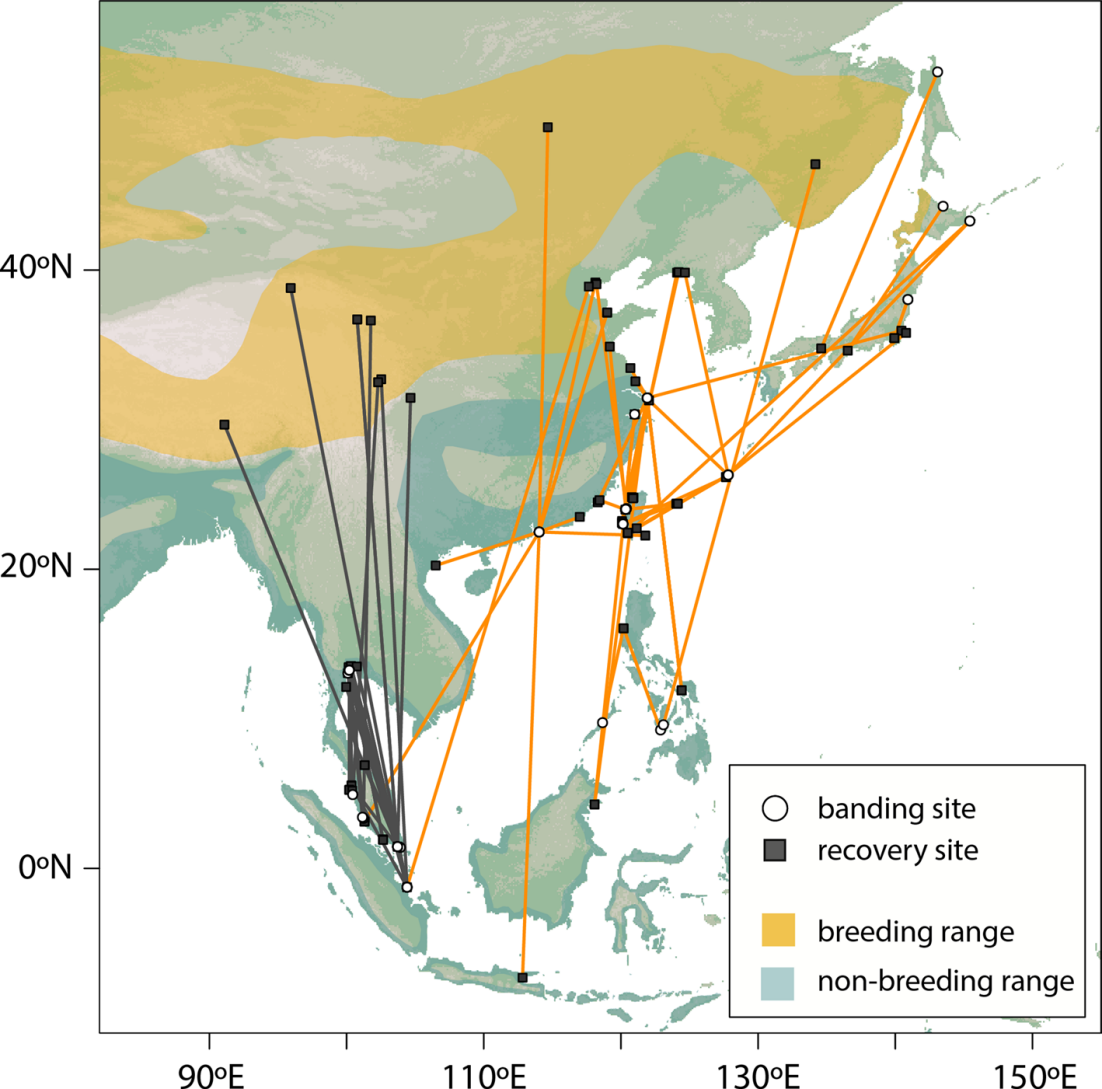
Migration routes of Common Redshanks wintering in Southeast Asia, based on ring recoveries and flag observations. Solid lines between banding and recovery sites are used to indicate the migration route. Black colour indicates birds breeding on the Qinghai-Tibet Plateau; Orange colour indicates birds breeding in East Mongolia, Northeast China and the Russian Far East. Data are derived from Supplementary Table S1. Map Source: ETOPO1 1 Arc-Minute Global Relief Model dataset provided by NOAA https://www.ngdc.noaa.gov/mgg/global/global.html. Species distribution map provided by BirdLife International.

Shorebirds wintering in Singapore and neighbouring Southeast Asian countries migrate predominantly along the East Asian-Australasian Flyway (EAAF), joining birds from Australia and New Zealand to breed mainly in Siberia and the Russian Far East^21,23–28^. However, some Curlew Sandpipers (*Calidris ferruginea*) have been documented migrating through Central Asia^28,29^ and some Common Redshanks (*Tringa totanus*) breed in the Qinghai-Tibet Plateau (located directly north of the Himalayas)^27,30,31^. Furthermore, Curlew Sandpipers, Sanderlings (*C. alba*) and Terek Sandpipers (*Xenus cinereus*), marked in the non-breeding season in Australia have been recorded during their migration period in the Indian sub-continent and Myanmar, just south of the Himalayas^12,32–34^. Such records indicate that some shorebird species wintering in Southeast Asia and Australasia could be migrating to breeding grounds in Central Asia, possibly crossing the Himalayas (as does the Brown-headed Gull, *Larus brunnicephalus* that winters in Thailand^35^). Singapore, situated near the equator just over 3000 km south-east of the eastern Himalayas, could be at the intersection between the CAF and the EAAF. For instance, records of Common Redshanks marked in Southeast Asia indicate that birds from the western region (e.g. Singapore and Malaysia) may migrate either to the Qinghai-Tibet Plateau adjacent to or beyond the Himalayas, or northeast towards breeding grounds from Mongolia to Russian Far East (Fig. 1, Supplementary Table S1).

In this study, we tested whether Common Redshanks and Whimbrels (*Numenius phaeopus*) from Singapore migrate across the Himalayas. We used light-level geolocators and satellite transmitters to track migrations of both species. Based on existing knowledge we expected that most individuals would migrate along more easterly routes but we aimed to (1) document whether any individuals made a more westerly crossing of the Himalayas; (2) determine whether any individuals avoided the Himalayas by making a detour around them; and (3) describe the relative migration patterns in terms of the general migration metrics, timings and stopovers for birds making trans-Himalayan flights versus those that did not.

## Results

### Migration routes

**Figure 2:**
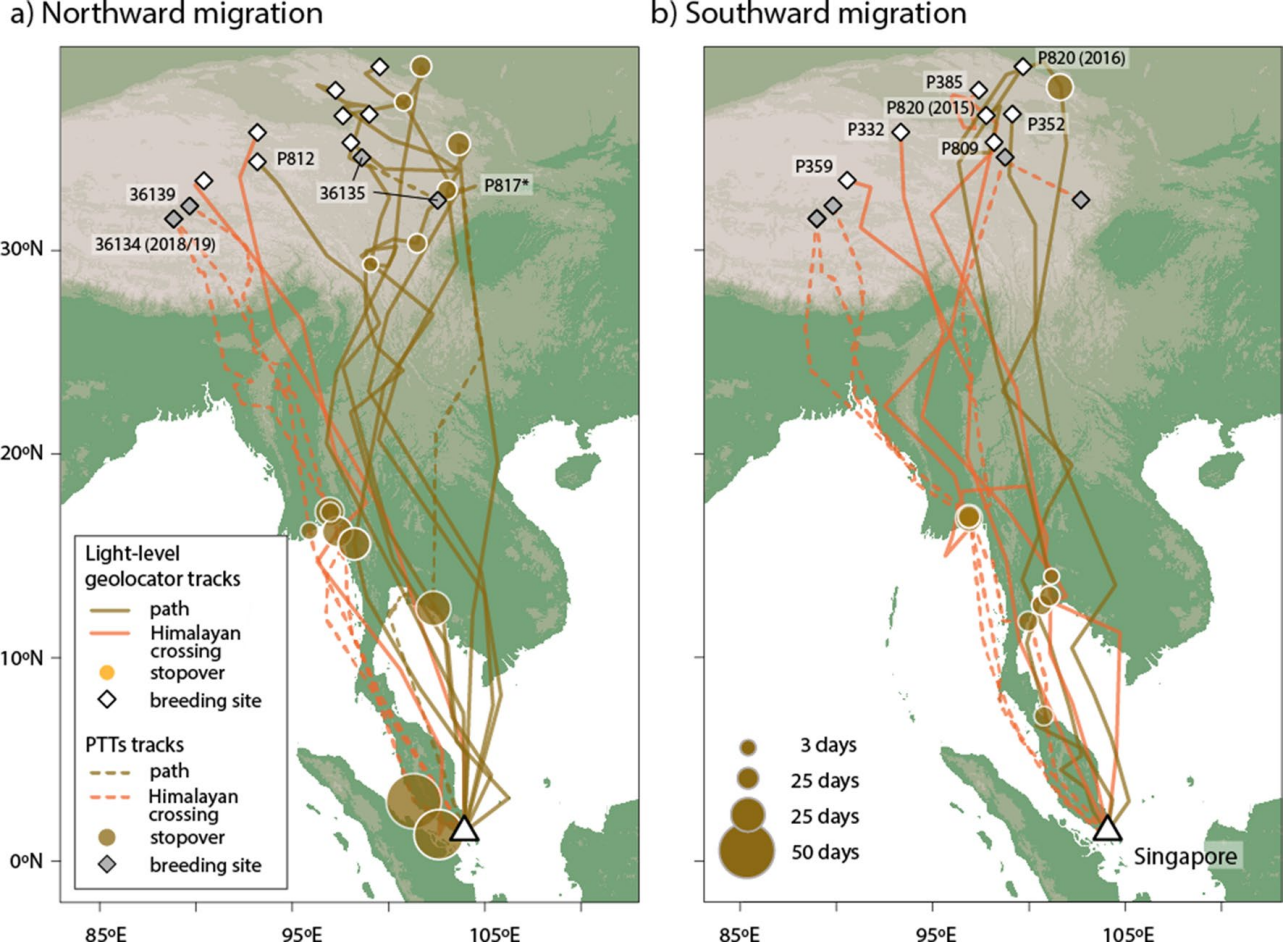
Migration routes of Common Redshanks based on light-level geolocation in 2015 and 2016, and PTT satellite transmitters in 2018 and 2019. Map Source: ETOPO1 1 Arc-Minute Global Relief Model dataset provided by NOAA https://www.ngdc.noaa.gov/mgg/global/global.html.

All ten Common Redshanks were tracked via the CAF to breeding grounds on the Qinghai-Tibet Plateau in China, but the northward migration routes taken varied from passing Myanmar in the west to near the Gulf of Tonkin in Thailand in the east (Fig. 2, Supplementary Fig. S1). Three birds crossed the Himalayan mountain range between longitudes 90.5° E and 96° E (Table 1) and bred in south-central Qinghai-Tibet Plateau (31–36° N, 89–94° E). The remaining seven birds ascended the scarp along the eastern edge of the Qinghai-Tibetan Plateau (between 97–105° E through Yunnan, Sichuan and eastern Xizang). They bred mainly in north-eastern Qinghai-Tibet Plateau (34–39° N, 93–103° E), apart from one bird that bred in the same general area as the easternmost trans-Himalayan migrant. Elevations of breeding areas were 4510–4570 masl for the more westerly trans-Himalayan migrants and 3520–4200 masl for the more easterly birds (based on satellite-tracking locations). On southward migration, the same three trans-Himalayan individuals returned via the Himalayas, but so did three of the eastern birds (Table 1, Supplementary Table S2); the remainder travelled south via Yunnan, Laos and Thailand. Birds taking a Himalayan route had a shorter migration on the way north than eastern birds did (4043 ± 303 km cf. 4702 ± 345; Table 2), and had a shorter detour (extra track length above the straight-line distance from non-breeding to breeding grounds: 241 ± 268 km cf. 831 ± 275 km; Z = 2.1396, P = 0.0226). As some eastern-breeders crossed the Himalayas on the way south, there was no significant difference in route length on southward migration.

**Table 1:** Summary of date, time and location of Common Redshanks and Whimbrels tracked undertaking trans-Himalayan crossings.

Species	Individual	Tag type	Northward migration		Southward migration	
			Date	Crossing location	Date	Crossing location
Common Redshank	P332	Geolocator	12/5/2015	93–96° E	25/8/2015	93–96° E
Common Redshank	P359/36139	Geolocator	20/5/2015	93–96° E	30/8/2015	93–96° E
Common Redshank	P359/36139	Satellite tag	19/5/2018	92.3–93.3° E	31/8/2018	91.2–91.7° E
Common Redshank	36134	Satellite tag	16–17/5/2018	91.5–92.5° E	28/8/2018	89.5–90.5° E
Common Redshank	36134	Satellite tag	13–14/5/2019	90.5–91.2° E	31/8/2019	88.5–89.2° E
Common Redshank	P385	Geolocator	n/a	n/a	6/7/2015	95–98° E
Common Redshank	P809	Geolocator	n/a	n/a	10/7/2015	94–97° E
Common Redshank	36135	Satellite tag	n/a	n/a	22/8/2018	96–97° E
Whimbrel	168750	Satellite tag	16–17/5/2018	91.5–93.5° E	20–21/9/2018	84–87° E
Whimbrel	168750	Satellite tag	13/5/2019	94–97° E	29–31/8/2019	77–82° E

All dates are indicated by day/month/year.

*n/a *not applicable.

**Table 2:** Summary of migratory movements of adult Common Redshanks traveling between Singapore and their breeding grounds as determined by light-level geolocation and PTT satellite transmitters.

Route	Northward migration							Southward migration					
	Date depart non-breeding grounds	#stopovers	Days at stopovers^a^	Date arrive breeding grounds	Days travelling	Migration distance N (km)	Days at breeding grounds	Date depart breeding grounds	#stopovers	Days at stopovers^a^	Date arrive non-breeding grounds	Days travelling	Migration distance S (km)
**All birds**													
Mean ± SD (n)	18/4 ± 19.4 (10)	1.0 ± 0.8 (10)	14.7 ± 19.5 (10)	8/5 ± 15.7 (9)	21.1 ± 20.7 (10)	4482 ± 454 (9)	95.0 ± 11.2 (8)	10/8 ± 24.8 (8)	1.1 ± 1.4 (8)	9.4 ± 11.4 (8)	26/8 ± 29.4 (7)	17.9 ± 13.7 (7)	4789 ± 1071 (6)
Range	13/3–9/5	0–2	0–62	16/4–22/5	3–70	3735–4999	74–105	2/7–3/9	0–4	0–31	23/7–2/10	4–42	3964–6571
**Trans-Himalayan**													
Mean ± SD (n)	19/4 ± 32.1 (3)	1.3 ± 1.2 (3)	24.0 ± 33.3 (3)	19/5 ± 4.9 (3)	29.7 ± 35.4 (3)	4043 ± 303 (3)	100.0 ± 2.6 (3)	8/8 ± 26.7 (6)	1.5 ± 1.4 (6)	12.5 ± 11.6 (6)	18/8 ± 32.8 (5)	22.4 ± 13.8 (3)	4679 ± 1264 (4)
Range	13/3–9/5	0–2	0–62	13/5–22/5	4–70	3735–4341	99–103	2/7–28/8	0–4	0–21	23/7–2/10	5–42	3964–4679
**Eastern**													
Mean ± SD (n)	18/4 ± 14.9 (7)	0.9 ± 0.7 (7)	10.7 ± 11.8 (7)	2/5 ± 16.7 (7)	17.4 ± 13.1 (7)	4702 ± 345 (6)	92.0 ± 13.7 (5)	16/8 ± 25.5 (2)	0 ± 0 (2)	na	23/8 ± 29.0 (2)	6.5 ± 3.5 (2)	5009 ± 885 (2)
Range	1/4–8/5	0–2	0–29	16/4–21/5	3–38	4116–4999	74–105	29/7–3/9	0–0	na	2/8–12/9	4–9	4383–5635
Z-value	− 0.1065	− 0.8452	− 0.9865	− 1.4721	− 0.8585	2.0516	1.3836	0.3873	− 1.3546	na	− 0.1993	− 1.3843	0.35609
P	0.8295	0.6635	0.411	0.1837	0.4865	0.0397	0.1035	0.8138	0.252	na	0.7609	0.1838	0.7968

Z-values and P-values are from permutation tests comparing trans-Himalayan and eastern migration routes.

All dates are indicated by day/month/year in SGT(UTC + 8).

^a^See methods. For the list of stopover sites see Supplementary Table S4.

**Figure 3:**
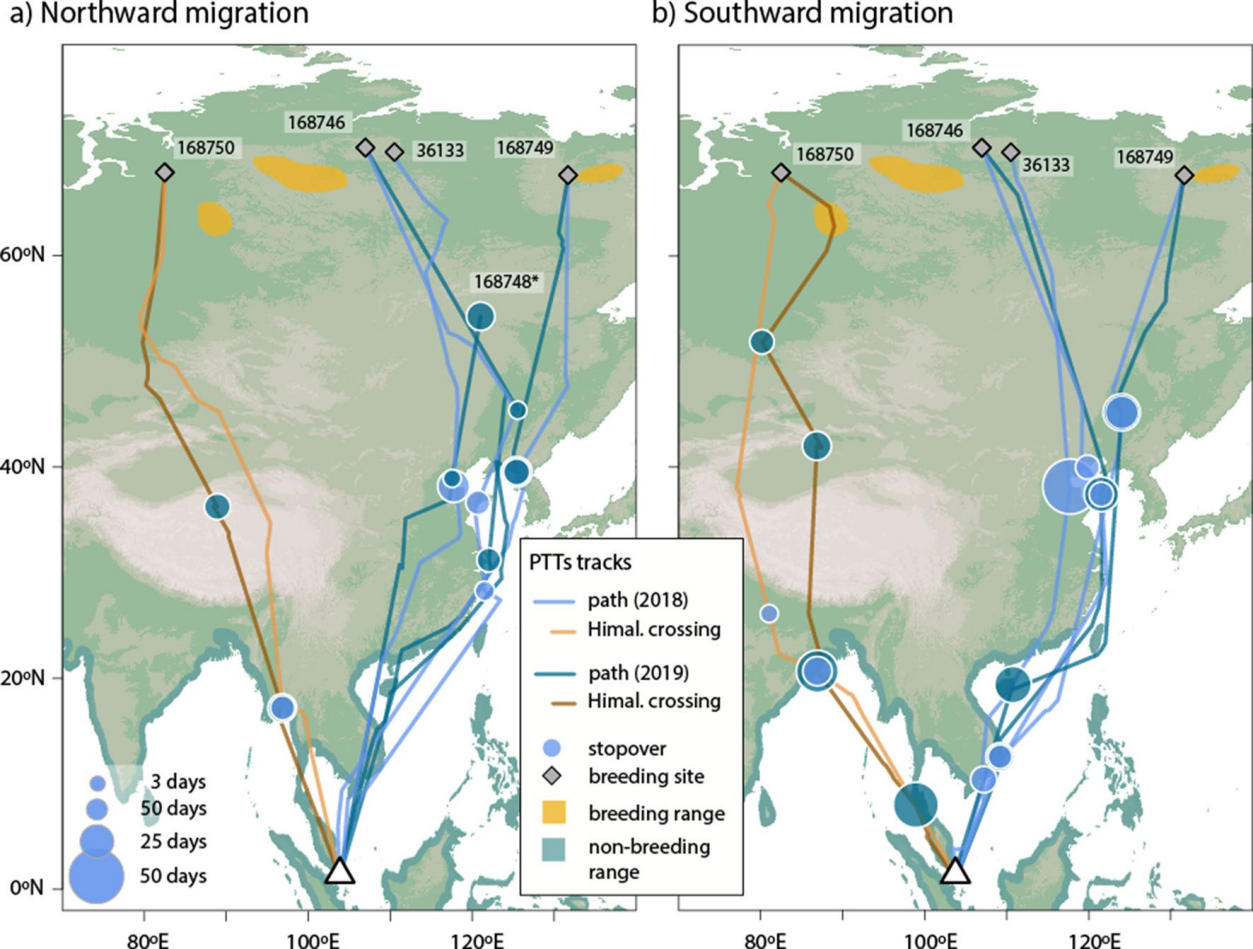
Migration routes of Whimbrels based on PTT satellite transmitters deployed in Singapore in 2018 and 2019. Map Source: ETOPO1 1 Arc-Minute Global Relief Model dataset provided by NOAA https://www.ngdc.noaa.gov/mgg/global/global.html. Species distribution map provided by BirdLife International.

Whimbrels, which breed at much higher latitudes in Russia, showed two very different pathways (Fig. 3, Table 3, Supplementary Fig. S2, Supplementary Table S3). Of the five tagged individuals, four migrated coastally or overland to the Yellow Sea region through the EAAF, then overland to breeding grounds east of Taimyr Peninsula. One bird, however, migrated across the Himalayas via the CAF to the Qinghai-Tibet Plateau and then to the area south of the Yenisei Gulf in Russia, south-west of Taimyr Peninsula. It crossed the Himalayas at 91.5–93.5° E in 2018 and 94–97° E in 2019 on northward migration, and 84–87° E in 2018 and 78–82° E in 2019 on southward migration (Table 1). While the Himalayan migrant tended to migrate for longer but not travel quite as far as eastern birds, the differences in migration metrics between these samples were not significant (Table 3). In addition to the one trans-Himalayan adult, two sub-adult birds took the same initial westerly route to the Ganges Delta in Bangladesh and Gulf of Mottama in Myanmar respectively in 2019, but returned to wintering grounds in Singapore without completing a full migration to the breeding grounds (Supplementary Fig. S2).

**Table 3:** Summary of migratory movements of adult Whimbrels traveling between Singapore and their breeding grounds as determined by satellite transmitters.

Route	Northward migration							Southward migration					
	Date depart non-breeding grounds	#Stopovers	Days at stopovers^a^	Date arrive breeding grounds	Days travelling	Migration distance N (km)	Days at breeding grounds	Date depart breeding grounds	#Stopovers	Days at stopovers^a^	Date arrive non-breeding grounds	Days travelling	Migration distance S (km)
**All birds**													
Mean ± SD (n)	26/4 ± 4.5 (6)	1.8 ± 0.8 (6)	21.7 ± 6.7 (6)	3/6 ± 8.8 (5)	39.0 ± 6.8 (5)	8416 ± 393 (5)	67.0 ± 9.3 (5)	9/8 ± 11.7 (5)	2.6 ± 0.9 (5)	53.8 ± 27.3 (5)	15/10 ± 33.7 (5)	67.2 ± 31.3 (5)	8530 ± 178 (5)
Range	21/4–3/5	1–3	14–31	24/5–15/6	33–50	8039–9019	61–83	29/7–26/8	2–4 (5)	28–100 (5)	24/9–14/12	40–121	8317–8764
**Trans-Himalayan**													
2018	24/4 (1)	2 (1)	31 (1)	15/6 (1)	50 (1)	8039 (1)	61 (1)	15/8 (1)	4 (1)	100 (1)	14/12 (1)	121 (1)	8619 (1)
2019	25/4 (1)	2 (1)	14 (1)	4/6 (1)	40 (1)	8114 (1)	83 (1)	26/8 (1)	2 (1)	28 (1)	5/10 (1)	40 (1)	8556 (1)
**Eastern**													
Mean ± SD (n)	27/4 ± 5.7 (4)	2.0 ± 0.8 (4)	21.3 ± 5.1 (4)	30/5 ± 7.4 (3)	35.0 ± 2.6 (3)	8647 ± 365 (3)	63.7 ± 3.1 (3)	1/8 ± 4.9 (3)	2.3 ± 0.6 (3)	47.0 ± 4.4 (3)	28/9 ± 4.2 (3)	58.3 ± 5.1 (3)	8493 ± 238 (3)
Range	21/4–3/5	1–3	16–28	24/5–7/6	33–38	8353–9019	61–67	29/7–7/8	2–3	42–50	24/9–2/10	54–64	8317–8764
Z-value	0.3238	0.7667	0.2150	− 1.348	− 1.6151	1.6697	− 0.9844	− 1.7905	− 0.8165	− 0.6826	− 1.3448	− 0.7754	− 0.9093
P	0.8650	0.6663	0.8983	0.2966	0.1002	0.091	0.696	0.0981	0.6984	0.7066	0.1026	0.6982	0.3755

Z-values and P-values are from permutation tests comparing trans-Himalayan and eastern migration routes.

All dates are indicated by day/month/year in SGT(UTC + 8).

^a^See methods. For the list of stopover sites see Supplementary Table S6.

### Migration altitudes and wind assistance

**Figure 4:**
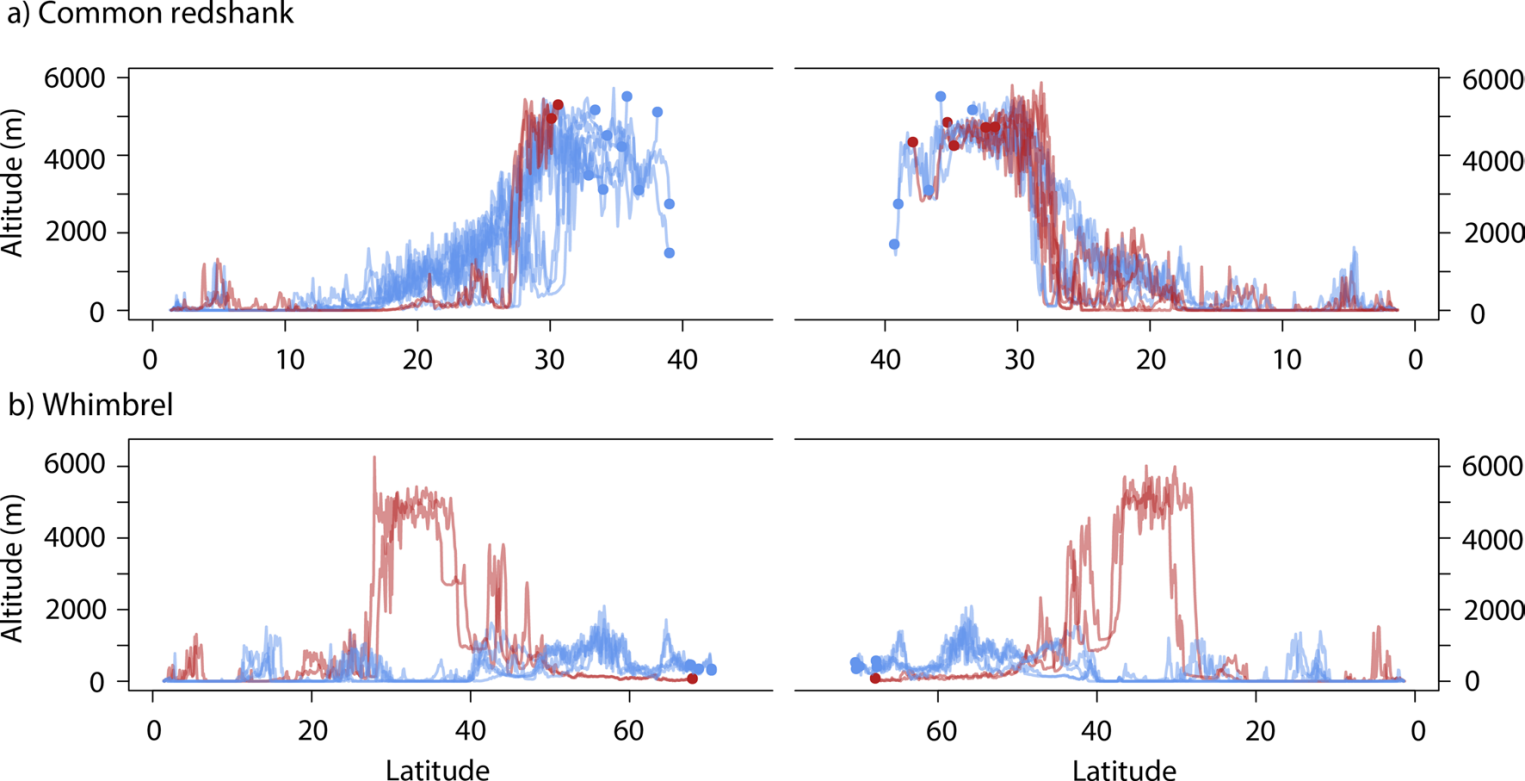
Latitude and altitude of northward and southward migration of adult Common Redshanks (n = 13, based on light-level geolocation and PTT satellite transmitters attached to birds in Singapore, 2015–2019) and adult Whimbrels (n = 8, based on PTT satellite transmitters attached to birds in Singapore in 2018 and 2019). Red lines indicate a trans-Himalayan route; blue lines indicate birds that migrated east of the Himalayan range (Common Redshanks) or through the East Asian-Australasian Flyway (Whimbrels).

**Figure 5:**
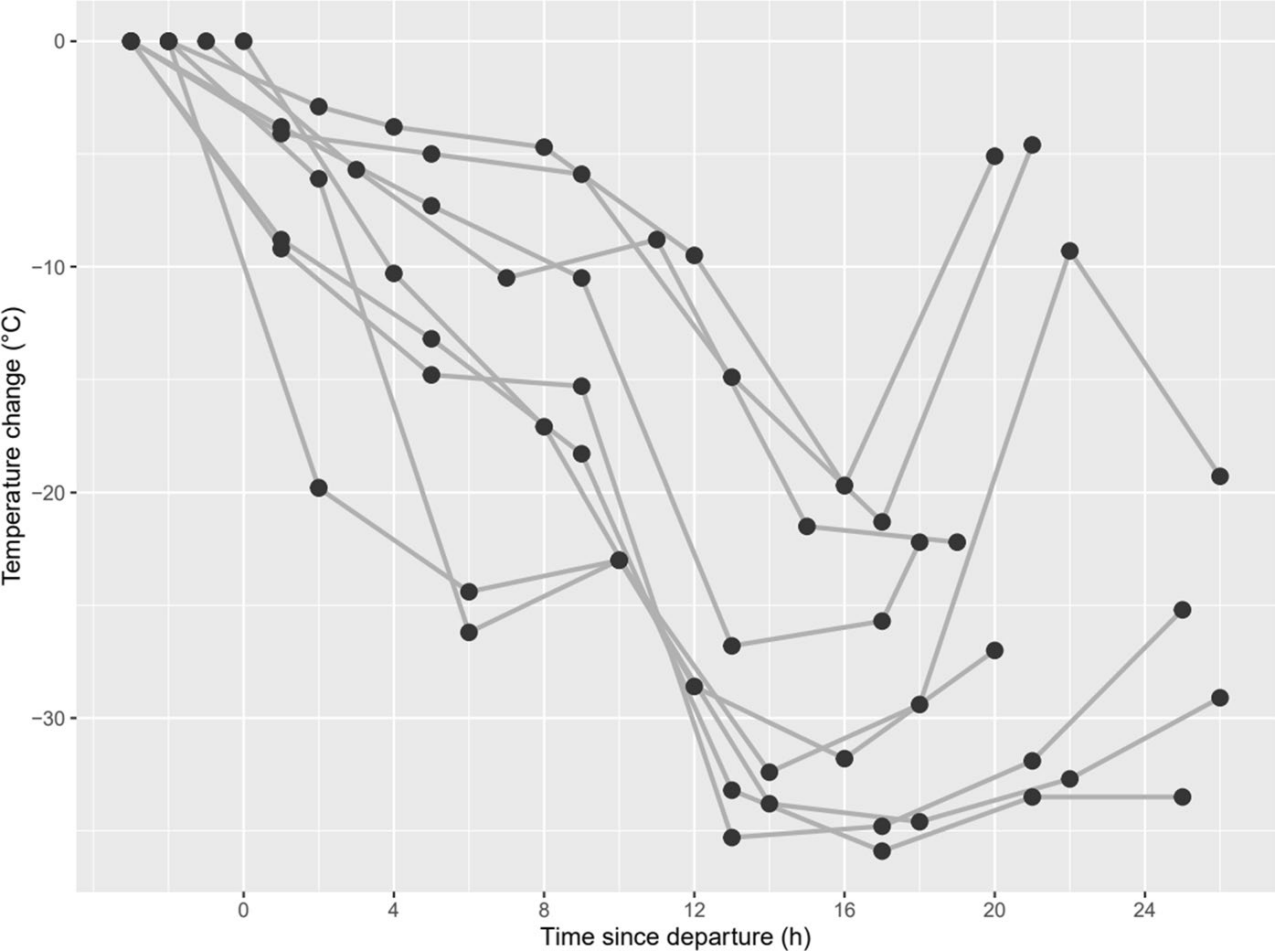
Temperature changes recorded by geolocators on Common Redshanks at the start of the initial northward flight from the tropical non-breeding grounds (nine records from eight birds).

Despite the Himalayan mountain range requiring a substantial elevation gain to cross, the minimum elevation profiles for Common Redshanks crossing the Himalayas or migrating further east showed that there were no significant differences in the average elevations for these routes on either migration (Fig. 4). Geolocator temperature data for Common Redshanks indicate that birds flew at high altitudes throughout the migration route. Within 13–19 h of leaving the tropical wintering grounds on northward migration, all Redshank geolocators showed decreases of 19.7–35.9 °C (Fig. 5). These suggest that birds were flying at altitudes of potentially 3000–5000 m assuming a lapse rate of 6.5 °C per km, despite not flying over high-altitude terrain at that time.

In contrast, for Whimbrels, the Himalayan route was significantly higher than the predominantly coastal EAAF overall (means of 977 ± 102 m for Himalayan compared with 316 ± 56 m for EAAF; Supplementary Table S5), but there was no difference in elevation between northward and southward migrations.

**Figure 6:**
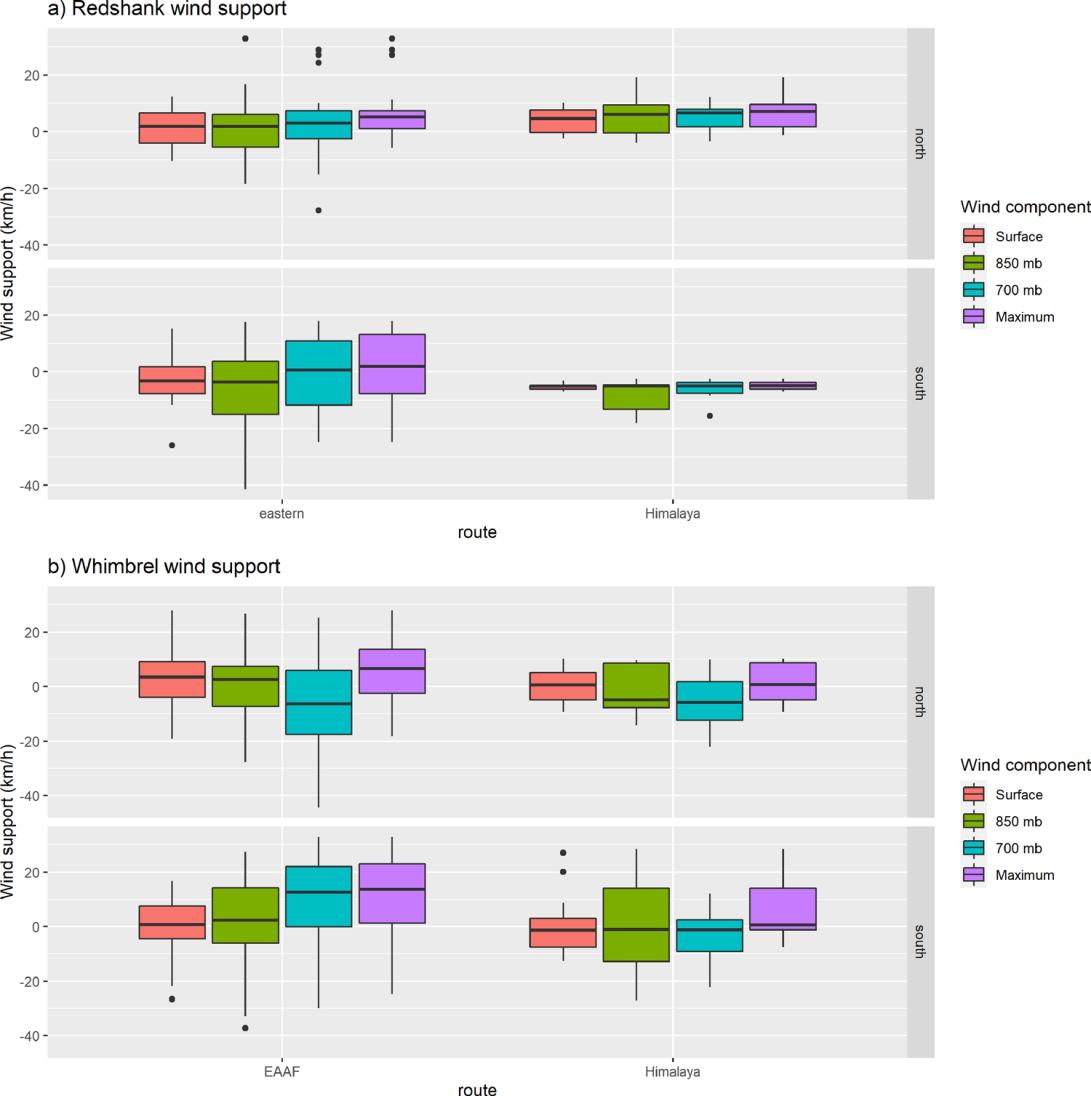
Wind support for Common Redshanks and Whimbrels migrating along trans-Himalayan or eastern routes from Singapore on northward and southward migration. Winds were estimated at three elevations along routes derived from geolocators (nine tracks of eight Redshanks in 2015 and 2016) or satellite transmitters (four tracks of three Redshanks and eight tracks of five Whimbrels in 2018 and 2019).

Both Common Redshanks and Whimbrels benefited from wind assistance during migratory flights. Data from satellite-tagged birds indicate that ground speeds increased, as wind support increased (for all air pressure levels tested—surface, 850 mb and 700 mb). However, air speeds decreased as the support increased (by 0.29–0.55 km h^−1^ for every km h^−1^ increase in wind support; Table 4). For Common Redshanks, there was no difference in wind support for Himalayan or eastern migrants, but there was less assistance on southward migration at surface and 850 mb (by 4.2–7.4 km h^−1^; Fig. 6; Table 5). For Whimbrels, wind support at high altitudes was considerably greater on southward migration than northward for migrants along the EAAF (by 12.8 km h^−1^), but was only marginally better for the Himalayan migrant heading south (Fig. 6; Table 5). Overall, ground speeds were very similar for the two species (Redshank, 55.1 ± 14.9 km h^−1^, range 29.0–94.8, N = 74; Whimbrel, 55.0 ± 15.5, range 21.4–93.2, N = 121, Supplementary Fig. S3) and there were no differences in ground speeds for birds when tracked crossing mountain ranges or not (Redshank, Z = − 0.014899, P = 0.9888; Whimbrel, Z = 0.60051, P = 0.5471).

**Table 4:** Effect of wind support on flight speed for Common Redshanks and Whimbrels.

Wind component	Ground speed				Air speed			
	F_df_	P	R^2^	Coefficient ± SE	F_df_	P	R^2^	Coefficient ± SE
**Redshank**								
Surface	23.23_1,64_	< 0.0001	0.37	1.145 ± 0.185	**0.648**_**1,64**_	**0.4238**	**0.01**	**0.146 ± 0.182**
850 mb	34.55_1,64_	< 0.0001	0.35	0.691 ± 0.118	6.585_1,64_	0.0126	0.09	− 0.287 ± 0.111
700 mb	19.48_1,64_	< 0.0001	0.23	0.567 ± 0.129	8.091_1,64_	0.0060	0.11	− 0.377 ± 0.133
Maximum	21.70_1,64_	< 0.0001	0.24	0.686 ± 0.147	4.527_1,64_	0.0372	0.07	− 0.314 ± 0.147
**Whimbrel**								
Surface	24.73_1,112_	< 0.0001	0.18	0.660 ± 0.133	6.706_1,112_	0.011	0.06	− 0.3392 ± 0.131
850 mb	22.70_1,112_	< 0.0001	0.17	0.456 ± 0.096	27.10_1,112_	< 0.0001	0.19	− 0.499 ± 0.096
700 mb	25.72_1,112_	< 0.0001	0.25	0.418 ± 0.083	44.55_1,112_	< 0.0001	0.28	− 0.551 ± 0.083
Maximum	24.76_1,112_	< 0.0001	0.18	0.545 ± 0.110	17.19_1,112_	< 0.0001	0.13	− 0.455 ± 0.110

Statistics are from linear regressions of ground speed estimated from short-term satellite-tracking estimates and calculated wind support. Overall regression statistics are provided, along with the coefficient of the relationship if significant. Coefficients represent the change in ground speed or air speed in km h^−1^ with an increase of 1 km h^−1^ in wind support. Non-significant models are shown in bold.

**Table 5:** Variation in wind support with migration route and direction for Common Redshanks and Whimbrels.

Wind component	Model			Route (Himalaya)	Direction (southward)	Interaction (Himalaya:south)
	F_df_	P	R^2^	Coefficient ± SE	Coefficient ± SE	Coefficient ± SE
**Redshank**						
Surface	3.34_3,62_	< 0.025	0.14	**1.745 ± 2.642**	− 4.180 ± 2.086	**− 4.833 ± 4.253**
850 mb	3.67_3,62_	< 0.017	0.15	**3.221 ± 4.209**	− 7.383 ± 3.324	**− 6.780 ± 6.775**
**700 mb**	**1.75**_**3,62**_	**0.167**	**0.08**	**1.424 ± 4.356**	**− 4.526 ± 3.441**	**− 6.893 ± 7.013**
Maximum	2.97_3,62_	0.0397	0.13	**− 0.379 ± 3.654**	**− 5.231 ± 2.886**	**− 6.410 ± 5.881**
**Whimbrel**						
**Surface**	**0.501**_**3,110**_	**0.682**	**0.14**	**− 2.058 ± 3.275**	**− 2.489 ± 2.245**	**2.300 ± 4.449**
**850 mb**	**0.354**_**3,110**_	**0.354**	**0.01**	**− 1.781 ± 4.583**	**2.503 ± 3.142**	**− 0.566 ± 6.226**
700 mb	7.155_3,110_	0.0002	0.16	0.525 ± 4.836	14.635 ± 3.156	− 12.828 ± 6.571
**Maximum**	**2.576**_**3,110**_	**0.0575**	**0.07**	**− 5.015 ± 3.860**	**5.113 ± 2.646**	**− 0.592 ± 5.243**

Statistics are from linear regressions of wind support in relation to route × direction. Overall model statistics are provided, along with the coefficients for factors. Coefficients represent the change in wind support in km h^−1^ when moving from eastern (reference) to Himalayan routes or from northward (reference) to southward migrations. Non-significant models or coefficients are shown in bold.

### Timing and stopover site use

**Figure 7:**
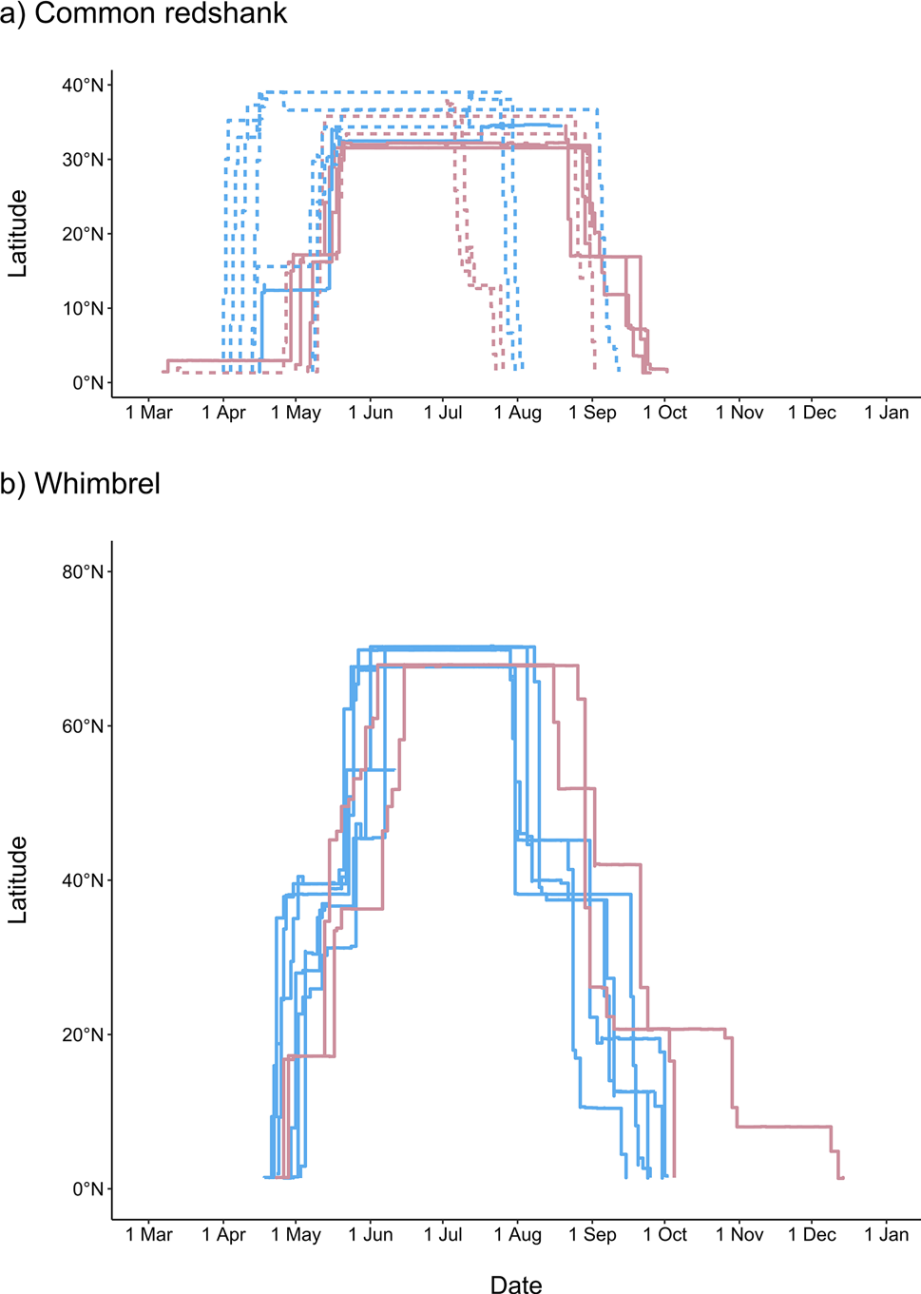
Timing and latitude of northward and southward migration of adult Common Redshanks and Whimbrels from Singapore. Migration chronology was estimated for birds tracked by geolocators (dashed lines; nine tracks of eight Redshanks in 2015 and 2016) or satellite transmitters (solid lines; four tracks of three Redshanks and eight tracks of five Whimbrels in 2018 and 2019). Red lines indicate birds that made a trans-Himalayan crossing; blue lines represent birds that migrated east of the Himalayan range (Redshanks) or along the East Asian-Australasian Flyway (Whimbrels).

Most of the Common Redshanks departed from Singapore between 1 April and 9 May (Table 2, Fig. 7a, Supplementary Table S2). They either migrated over a short duration (3–4 days), or made one or two stopovers and took a duration of 13–32 days to reach the breeding grounds. Stopovers were defined as sites that birds used for at least 3 days (see Supplementary Table S4). The exception was one bird, which migrated north in early-mid March both in 2015 and 2018. It made prolonged stopovers and took 70 and 72 days, respectively to reach the breeding grounds. The variation in migration pattern was not strongly associated with migration route. Both trans-Himalayan and eastern migrants included birds that made stopovers and others that flew directly to the breeding grounds. While individuals that crossed the Himalayas arrived slightly later at their breeding grounds than eastern birds (Table 2, Fig. 7a), there was overlap in timing and the differences were not significant. Three Common Redshanks were tracked in two years (via geolocator, satellite transmitter, or geolocator + satellite transmitter) and showed highly consistent migration timing, with differences of 4–5 days in departing Singapore, 3–4 days in arriving on the breeding grounds and 2–3 days in leaving the breeding grounds (Supplementary Table S2). The duration of northward migration was correlated with the duration of southward migration for most individuals (R^2^ = 0.83, P = 0.0112); the exception was a trans-Himalayan migrant that made two stopovers and took 70 days to migrate north, but travelled directly back to the non-breeding grounds in 5 days. The duration of southward migration appears shorter for eastern than trans-Himalayan migrants (6.5 versus 22.4 days). However, the eastern sample included only two birds, both of which were migrated over a short duration on the way north (4 and 9 days). Tracked Common Redshanks reached the non-breeding grounds from late July to early October (Table 2).

The Whimbrels departed from Singapore within a period of 14 days (20 April–3 May), with differences between years for the three birds tracked in successive years of only 1 or 2 days (Table 3; Fig. 7b). The four individuals migrating through the EAAF made 1–3 stopovers (Supplementary Table S6) totaling 16–28 days and took 33–38 days to reach the breeding grounds in late May or early June. The bird that crossed the Himalayas to western Siberia made 1–2 stopovers (Supplementary Table S6) totaling 14–31 days and took 40–50 days to reach the breeding grounds in early-mid June. In addition to taking a longer duration to reach its breeding grounds, this individual remained on the breeding grounds slightly later than EAAF migrants (15 and 26 August cf. 29 July–7 August; Table 3). Southward migration took significantly longer than northward, with EAAF birds taking 16–31 days longer on the way south and the Himalayan bird taking 121 days to reach the non-breeding grounds in one year (permutation test for all birds, Z = − 20.35, P = 0.0041; for just the EAAF birds, Z = − 2.7939, P = 0.0092). Most Whimbrels reached the non-breeding grounds from late September to early October (Table 3) except for the trans-Himalayan migrant when it made stopovers in China, India and Thailand (Supplementary Table S6) and only arrived back in mid-December.

## Discussion

### Singapore at the intersection of the EAAF and CAF

All ten Common Redshanks and one of the five Whimbrels tracked from Singapore migrated along a westerly route through the CAF, in contrast to previous knowledge that shorebirds wintering in Southeast Asian countries migrate predominantly along the EAAF^21,23,27,28^. To undertake this migration route, birds used two strategies to deal with the barrier posed by the Himalayan range particularly during the northward migration; with three Common Redshanks and one Whimbrel making a direct crossing, and seven Common Redshanks avoiding the Himalayas by ascending the plateau to the east through Yunnan and Sichuan, possibly helping their ascent of the Qinghai-Tibet Plateau by using valleys along major rivers of this region. During southward migration, all four birds (three Common Redshanks and one Whimbrel) and an additional three Common Redshanks directly crossed the Himalayas. This is the first time that shorebird migration routes across the Himalayas from Southeast Asia have been documented.

It is to be noted that the sub-adult Whimbrels and first year Common Redshank did not migrate to the breeding grounds (Supplementary Figs. S1 and S2). This is consistent with the prevailing view^36^ that young birds do not make complete migrations before they participate in breeding. While “over-summering” of young birds on the non-breeding grounds is common, particularly in large shorebird species^37^ in which delayed maturity may be prolonged^38^, demonstrations of partial migration by tracking are rare.

The results (Figs. 2 and 3) and the supplementary ring recapture and flagging resighting data (Fig. 1) indicate that Singapore is at the intersection of the EAAF and the CAF. Some Common Redshanks from the Thai-Malay Peninsula, and all those from the eastern parts of Southeast Asia (East Java, Borneo and the Philippines) take an easterly migration route along the EAAF through the South and East China coast and Japan to breed in a zone from East Mongolia to the Russian Far East (Fig. 1, Supplementary Table S1). Most Whimbrels that we tracked from Singapore took a similar route to birds from Australia that migrated through coastal east China to breed in the Russian Far East^24,39^. The Whimbrel that crossed the Himalayas probably represents a different breeding population than those migrating along the EAAF, whereas for Common Redshanks, those crossing the Himalayas tended to be the more westerly breeders within one population.

### Making the trans-Himalayan crossing

The direct crossing of the Himalayas requires shorebirds to fly at a great altitude. While the highest peaks in the Himalayas exceed 8000 masl, the lowest passes available in the eastern Himalayas are at 4225 masl (Sela Pass) and 4310 masl (Nathu Pass). As we do not have in-flight measurements of the actual altitude of birds during migration, we cannot confirm the height at which birds crossed the Himalayas. However, ground elevations based on tracks indicate that all Common Redshanks likely reached 4800–5800 m at some stage in their migrations en route to breeding grounds at 4510–4570 masl. The migration routes of satellite-tagged Common Redshanks indicate they use slightly different locations to cross between years and during northward migration versus southward migration. Therefore, Redshanks must be able to fly at altitudes of more than 5000 masl to make a trans-Himalayan crossing.

The one Whimbrel tracked to the Qinghai-Tibet Plateau had a much wider range of Himalayan crossing points than did Common Redshanks, using the eastern Himalayas during northward migration and central to central-west Himalayas during southward migration. The northbound stopover site used in 2018 was at 4950 masl, while the southward migration in 2019 required the bird to cross both the Kunlun Mountains and the Himalayas, and the Qiangtang Plateau between them, with an average elevation of 5000 masl. The lowest passes available are about 5000 m in the west-central Kunlun Mountains (Mazah Pass at 4969 masl and Sanju Pass at 5364 masl) and the only pass lower than 5200 m in central to central west Himalayas is 4720 m (Shiphi La). This suggests that Whimbrels have to fly at altitudes of 5500–6000 masl to be able to migrate along this route. These findings support the view of Prins and Namgail that there are no feasible routes to cross the Himalayas and Tibetan Plateau for birds that cannot fly higher than 5000 masl^13^.

While the elevation profiles for Himalayan and eastern migration routes (Fig. 4, Supplementary Table S5) showed that Himalayan migrants must fly across high elevation, for Whimbrel this was only transient en route to lowland breeding grounds in north-central Russia. For the Common Redshanks, the western ascent via the Himalayas was steeper than the ascent via the eastern route. Notwithstanding this, the average elevation did not differ between the Himalayan route or eastern route. If high altitude flight is the major physiological challenge of these journeys, there is evidently little difference in the challenge for Himalayan versus eastern routes, at least for the Common Redshanks. This is consistent with known records of shorebirds capable of migrating at high altitudes, e.g. shorebirds have been recorded flying at altitudes of 5800 masl when crossing the Pamirs^40^ and Black-tailed Godwits (*Limosa limosa*) have been recorded flying at nearly 6000 masl on migration south from the Netherlands^41^. Our temperature data from geolocators indicate that Redshanks likely flew at 3000–5000 m altitude even when not facing mountains of that height (Fig. 5), similar to the altitudes observed in migrating Arctic shorebirds^42^. Given the propensity for shorebirds to fly at high elevations, the Himalayas may not present an insurmountable barrier, as long as they can avoid the highest peaks. This is corroborated by the observations of trans-Himalayan specialists, such as the Bar-headed Goose, *Anser indicus*^18^.

Consistent with other studies^1,5,7,41,43^, satellite-tagged birds tended to migrate with wind assistance. The decrease in calculated airspeed with wind support indicated that birds reduced their ground speed (and energy expenditure) when winds were favourable. The temperature decrease shown by Common Redshanks geolocators indicated flight altitudes of 3000–5000 m. This in turn suggested that birds sought tailwinds at higher altitude, although this could also be attributed partly to an avoidance of tropical air temperatures^41^. However, birds did not always have tailwinds. Headwinds predominated during southward migration for Common Redshanks, as southerly winds in the region last from May until October/November. For Whimbrels, tracking data suggested that high-altitude winds were greater on southward migration, but mostly for EAAF migrants. The trans-Himalayan crossing did not seem to be assisted by especially favourable winds, compared with those using an easterly route, though we do not have detailed positional and wind data from across multiple routes.

Common Redshanks are coastal in the non-breeding season, but the tracked birds bred at altitudes that were not much lower than the altitudes at which they migrated across the Himalayas (5000–5500 masl; Fig. 4). The physiological adaptations to flight and life at high altitude with thin air and low oxygen levels have been well studied in Bar-headed Geese and Andean Geese (*Chloephaga melanoptera*)^44,45^. Common features of birds that migrate at high altitudes include more effective gas exchange with greater hypoxic ventilatory response, larger lungs with higher capillarization, enhanced cardiac output, greater oxygen delivery to skeletal muscles due to increased haemoglobin oxygen affinity, and higher capillary to fibre ratios with increased oxidative capacity compared to low altitude migrants^45,46^. Migratory birds are notable for their physiological flexibility during migration, including anticipatory increases in mass, organ sizes and modulation of underlying biochemistry^47–49^. These changes are evidently sufficient to enable shorebirds to embark on high altitude flights directly from coastal fuelling sites. Some Common Redshanks taking more easterly routes did make stopovers at altitude that might have allowed some acclimation to high altitude conditions, but that is evidently not essential as some individuals flew direct to the breeding grounds on both Himalayan and eastern routes (Fig. 2).

### Migration timing

The wide range of migration timings in Common Redshanks was unexpected (Table 2, Fig. 7), with almost two months between the first and last departures on northward migration. This variation arose through a mix of migration strategy (direct versus stopovers) and breeding destination. The individual that departed its non-breeding grounds earliest, in early-mid March, took 70–72 days to reach its breeding grounds, but showed only 5 days difference in departure date between years. The consistency shown in the Common Redshanks that have been tracked in both years (Supplementary Table S2) implied that the large inter-individual variation in timing was real.

### Migratory trade-offs

On balance, the benefit of a Himalayan route is that it is a shorter migration distance to the western part of the breeding range. Detouring around the Himalayas would add around 600 km to the migration distance. Location of an individual bird’s preferred breeding site is therefore likely to be an important determinant in the choice of migration route. There is, however, one way in which the trans-Himalayan and more eastern routes taken by Common Redshanks may differ in risk. That is in the ability of birds to approach the breeding grounds in a staged fashion via stopovers and evaluate local conditions. Eastern birds tended to make stopovers in China after reaching the Qinghai-Tibet Plateau (Fig. 2) whereas Himalayan migrants made coastal stopovers in Myanmar, over 1500 km from the breeding grounds. Trans-Himalayan individuals tended to remain at lower latitudes later than eastern migrants, and then flew directly across the Himalayas to the breeding grounds. As the potential costs associated with crossing the Himalayas and finding the Qinghai-Tibet Plateau still frozen must be high, it is likely that there is more flexibility allowed in travelling along the eastern route compared with the Himalayan route. Trade-offs are implied between migration routes and risks.

### Conservation implications

Our findings of Common Redshanks and Whimbrels migrating along a westerly route across the Himalayas indicated that shorebirds wintering in Southeast Asian countries used both the CAF and EAAF to reach their breeding grounds. The current conservation initiatives, such as the East Asian-Australian Flyway Partnership, may need to pay greater attention to the conservation of stop-over and breeding sites along the westerly route. In particular, the Gulf of Mottama (formerly known as Martaban) appears to be the last important refuelling site for Southeast Asian shorebirds undertaking the energy-demanding trans-Himalayan flight. Two Common Redshanks and one Whimbrel used the Gulf for 9–20 days during northward migration, while two Common Redshanks used the area for 11–21 days during southward migration. The full lists of stopover sites in Supplementary Tables S4 and S6 provide a first set of conservation targets.

Even more important for conservation, the CAF route may be commonly used by other shorebirds, such as Lesser Sand Plovers (*Charadrius mongolus*) that commonly breed on the Qinghai-Tibet Plateau^50,51^; four records of tagged individuals from the Thai-Malay Peninsula have been obtained in Myanmar and one in Sichuan (Supplementary Table S7). Other likely species include Asian Dowitcher (*Limnodromus semipalmatus*), Curlew Sandpiper, Sanderling and Terek Sandpiper^12,32–34^ (Supplementary Table S7), as individuals of all these species tagged in Australia and the Thai-Malay Peninsula have been recorded in South and Central Asia. However, the full migration routes of all these species have yet to be studied by satellite tracking or geolocators.

### Return rates from tracking device

The return rates of marked shorebirds at Sungei Buloh Wetland Reserve were slightly lower for adult Common Redshanks with geolocators at 66.0% (n = 97), 63.8% (n = 58), 63.9% (n = 36) and 71.4% (n = 21) from the 1st to 4th years after tagging, compared to adult birds with flags only in the same year at 72.3% (n = 159), 79.3% (n = 121), 74.2% (n = 97) and 72.6% (n = 73) respectively. Sample sizes of satellite tagged Redshanks were too small to reliably evaluate return rates [which were 50% (n = 4), 50% (n = 2) and 100% (n = 1) from the 1st to 3rd years after tagging]. The resighting of satellite tagged Whimbrels from SBWR gave a 100% return rate for the 1st (n = 2) and 2nd year (n = 5) after tagging as compared to non-satellite tagged birds at 96.3% (n = 27) and 81.5% (n = 27) in the same year.

Overall, our study provided first-hand information on the strategy of shorebirds undertaking a remarkable journey across the Himalayas. Gaps in our knowledge remain on the details of this migration strategy, including exact altitudes used and physiological adaptations for high-altitude flight and breeding. Using improved tracking devices of lighter weight and greater accuracy, including altitude data during migration and more frequent transmission will help to better understand the trans-Himalayan strategy of small to medium-sized shorebirds in the future.

## Methods

### Ringing and flagging data

We collated and analysed bird ringing recoveries and flag observations, including both published and unpublished data contributed by the Australasian Wader Studies Group (AWSG) and major bird ringing centres in Asia as well as individual birdwatchers (see acknowledgements) to prepare Fig. 1, Supplementary Tables S1, S7.

### Bird capture and aging

We used geolocators and satellite transmitters to investigate the migration routes of shorebirds that wintered in Singapore. Both were affixed primarily on adults (birds > 1 year old) since first-year shorebirds do not usually migrate to breeding grounds^36^. Birds were caught in mistnets at night at Sungei Buloh Wetland Reserve (SBWR, 1.45° N, 103.73° E) and Chek Jawa Wetlands (CJ, 1.41° N, 103.99° E) in Singapore following established bird ringing protocols (NParks SBWR, unpublished) to ensure the safety of the birds. Birds were ringed with a metal ring on the tarsus and given two plastic leg flags (green/white) on the right tibia. Birds were aged on plumage characteristics, distinguishing between first-years and birds older than 1 year (by retained juvenile feathers, or by primary feather moult as first-year shorebirds in Singapore normally do not moult their primaries, some Common Redshanks may only moult their outer primary). Birds older than first-year were not able to be aged further. One Whimbrel (168747) tracked as a first-year bird (and therefore of known age) did not migrate in the first two years and conducted only a partial migration in the third year. Two other Whimbrels that also made only partial migrations were assumed to be sub-adult birds. The first-year and sub-adult birds are not included in the migration statistics or figures. A study in New Zealand suggests that immature Bar-tailed Godwits (*Limosa lapponica*) wintering there ranged widely within the country during the species’ usual breeding season, before embarking on their first northward migration at age 2–4 years^38^.

Geolocator tracking requires the recapture of tagged birds for data recovery and processing^52,53^. Therefore, we chose to study Common Redshanks that are relatively faithful to the same non-breeding sites^54,55^. A total of 99 geolocators (model: Intigeo-C65, Migrate Technology Ltd., UK), each weighing 1 g, were deployed on Common Redshanks (97 adults, 2 first-year birds) between 24 October 2014 and 5 March 2015. Each geolocator was glued to a handmade leg flag that was attached to the left tibia of the bird. We retrieved 10 geolocators from Common Redshanks recaptured between September 2015 and December 2017 at SBWR (Supplementary Table S8). Recapture effort was stopped after a total sample size of 10 was reached to minimise disturbance. Geolocator tracking data were collected for eight adults in 2015, with further data for one individual (P820) collected in 2016; one geolocator did not record any data.

Light-intensity data were recorded at 5 min intervals and analyzed using a threshold method^56^. Sunrise and sunset events were identified on log transformed light data and a threshold of 1, using the R package TwGeos^57^. To define the error distribution of sunrise/sunset times caused by shading (e.g., clouds, habitat) and calculate the individual reference sun elevation angle needed for calibration, we used light recordings when the individual was at the known deployment site at the beginning or end of the time series. We then analysed the sunrise and sunset times to identify periods of residency (e.g., periods with no detectable movement). We considered birds to be residents during periods when consecutive twilight events changed only within the expected error range. Twilights were grouped surrounding twilights with a probability of movement > 0.5; periods of residency smaller than 2 days were discarded (for details on the method see Lisovski et al.^58^). The defined twilight times, the error distribution (gamma density distribution) and the grouping results were then used within the R package SGAT^59^ to refine track estimates using the grouped threshold method^56^. SGAT provides a Bayesian framework that allowed us to combine prior information on twilight error distribution and the flight speed distribution (defined using a relaxed gamma distribution of shape = 2.2 and rate = 0.08) with the location estimates. This allowed us to refine locations based on Markov chain Monte Carlo (MCMC) simulations and provided a probability distribution around each estimate (one location per period of residency and two locations per day for periods of movement). The first and last location (in case the logger was still recording light on return) were fixed to the deployment site. We first ran a *modifiedGamma* model (relaxed assumptions) for 1000 iterations to initiate the model, before tuning the model with final assumptions/priors (three runs with 300 iterations). Finally, the model was run for 2000 iterations to ensure convergence. We then used the last 2000 MCMC chains to estimate the most likely track (median location estimates) and the 95% credible intervals. We used the median locations for statistics and maps in the main paper and provided the 95% credible intervals in stopover and breeding site locations in Supplementary Table S4 and Supplementary Fig. S1.

Geolocators collected temperature data in 4-h blocks, but they also record wetness counts (capped at 14) in each hour within that block. We used the conductivity and wetness counts to determine the change in geolocator temperature in relation to time since departure, assuming that birds departed after the last hour showing wetness > 0^60^.

### Satellite tracking

Satellite tracking was conducted on 4 Common Redshanks and 5 Whimbrels at SBWR and 6 Whimbrels from CJ (Supplementary Table S8). Five gram solar-powered Platform Transmitter Terminals (Solar 5 g PTT, Microwave Telemetry, Inc., USA) were attached to Common Redshanks using a leg-loop harness^61^ on birds caught at SBWR between 16 October 2017 and 9 February 2018. Five gram and 9.5 g PTTs (Solar 9.5 g PTT, same company) were attached to Whimbrels using the same method during the non-breeding period at SBWR and CJ between 31 March 2017 and 5 December 2018.

Satellite tracking data, including time and location data were provided by Collecte Localisation Satellites through the Argos website (www.argos-system.org). Data were successfully obtained from three adult Common Redshanks in 2018; one tag continued transmitting through 2019. One Common Redshank (P359/36139) was tracked with both a satellite transmitter in 2018 and a geolocator in 2015. Satellite tracking data were successfully obtained from four adult Whimbrels in 2018; three of these individuals’ trackers also provided data into 2019. In addition, satellite tracking data were obtained from one adult and three sub-adult Whimbrels in 2019.

Satellite transmitters and their harnesses were well within the 3% body weight of whimbrels as suggested by Phillips et al.^62^ to minimize the impact on migration. We attached satellite transmitters to Common Redshanks that had a body mass > 120 g, making the transmitter approximately 4% of body mass. Shorebirds build up body mass before migration^63^ and SBWR ringing data has shown that individual Common Redshank can increase from 113 g in October to as much as 154 g in April just before migration (NParks SBWR, unpublished). Therefore, the transmitter was likely within 3% of the body weight at the time of migration.

The 9.5 g PTTs were set to operate 10 h on and 48 h off to ensure the batteries were continually charged for long term monitoring. The 5 g satellite transmitters had the same on/off time settings but also allowed data transmission when the transmitters were fully charged (regardless of duty cycle). Movement data were downloaded from the Argos website (www.argos-system.org). We used mainly Class 1 (accuracy between 500 and 1500 m), Class 2 (accuracy between 250 and 500 m) and Class 3 (accuracy within 250 m) data for analysis and mapping. However, we used less accurate data than Class 1 when the birds were moving to allow us to calculate more accurate departure and arrival dates, actual flight path (e.g. during migration and Himalayan crossing) or analyse speed and wind.

### Stopover sites

As PTTs were set to operate 10 h on and 48 h off, it is likely that stopovers shorter than 3 days will not able to be identified. Therefore, we identified stopover sites from the tracks as locations that were within the approximate error range of 150 km (inferred from variation of locations at the deployment site) for at least 3 days, preceded and followed by directional movements > 150 km. Short stopovers are also hard to reliably detect in geolocator data, given the error structure of the defined twilight times. Thus, we also used the 3-day threshold to indicate reliable stopover sites.

### Migration distances and ground speeds

Ground speeds and wind data for Whimbrels and Common Redshanks were only calculated for satellite tracked individuals during periods of transmission while the bird was moving. As the PTTs were programmed to a schedule of 10 h on and 48 h off, it’s not possible to calculate the full flown distance of the tagged birds. We used the great circle distance between consecutive locations and stopover sites between non-breeding and breeding grounds for both geolocator tagged birds and satellite-tagged birds to calculate the minimum flight distance birds took. Track distance and corresponding flight speed were calculated using the package “move” in R^64^. Speed error was included by summing the maximum error radius of two locations divided by the flying time between locations. As the PTTs were programmed to a schedule of 10 h on and 48 h off, apparent low speeds could include undetected short stopovers; we therefore excluded any apparent speeds of < 20 km h^−1^. In total there were 74 estimates of ground speed from three Common Redshanks and 121 estimates from five Whimbrels.

### Wind data

Wind speed and direction at surface (10 m above ground), 850 mb (~ 1500 masl) and 700 mb (~ 3000 masl) were obtained from the ERA-Interim dataset provided by the European Centre for Medium-Range Weather Forecasts (ECMWF) by bilinear interpolation, using the Env-DATA Track Annotation Service in Movebank^65^. We calculated wind support for all flight segments following Safi et al.^66^ and used v_w_ × cos(α) where v_w_ is wind speed and α is the difference between the bird’s track direction and wind direction. Increasing positive wind support values indicate stronger tailwinds, while increasing negative values indicate greater headwind conditions.

To analyse the effect of wind assistance on ground speeds we used linear models of ground speed versus wind assistance (including the maximum wind support at any level) to test whether ground speed varied with wind assistance for each species separately, treating each measurement as an independent data point. We also tested whether wind support at each altitude was different for birds crossing the Himalayas or on eastern routes and on northward or southward migration in linear models of wind support versus route + direction.

### Minimum track altitudes

Minimum elevations (masl) were determined at every 0.1°N along birds’ migration routes and averaged for each individual (north or south); these average values were tested for differences between trans-Himalayan and eastern routes by permutation tests (see below) conducted separately for the two species and for northward and southward migrations.

### Statistics

For Common Redshanks with 2 years of tracking data, we analysed migration metrics using the first of those years, though we present all tracking data in the visual summaries. One Whimbrel was tracked in 2 years; as this was the only Himalayan migrant, and showed a large difference in phenology on southward migration between years, we included both years of data in analyses.

As we have limited sample sizes within our study species and migration routes, for comparison between groups we used the oneway_test permutation test in the R-package “coin”, with 9999 MonteCarlo resamplings.

All means are presented ± SD.

### Return rate for impact assessment on tracking device

We used resighting data of engraved-flagged shorebirds at SBWR to assess the return rate of shorebirds (NParks SBWR, unpublished). The return rate of birds with flags only was compared with the return rate of geolocator-tagged and satellite-tagged birds. Recaptured redshanks whose geolocators were removed were treated as flagged only for subsequent return rates.

### Map and source

To illustrate the tracks and the ring recovery data on a map with a topographical profile we used the ETOPO1 1 Arc-Minute Global Relief Model dataset provided by NOAA^67^. Species distribution maps were provided by BirdLife International^68^. All maps were produced using R version 3.6.1^69^.

### Ethics statement

Research protocols were approved by National Parks Board, Singapore. All field work was conducted in accordance with regulations of the Parks and Trees Act and the Wild Animals and Birds Act.

## Supplementary information

Supplementary Information.

## Data Availability

Geolocator data including the raw light recordings, the annotated twilight files, the location estimates with credibility intervals and the R code will be uploaded onto Movebank upon paper acceptance. All data will be made available on reasonable request.
