# Supplementary material for: Shorebirds wintering in Southeast Asia demonstrate trans-Himalayan flights
Source: Sci Rep. 2020 Dec 11;10:21232. doi: 10.1038/s41598-020-77897-z (PMC7732824; doi:10.1038/s41598-020-77897-z)

Shorebirds wintering in Southeast Asia demonstrate trans-Himalayan flights

David Li[1], Geoffrey Davison[1], Simeon Lisovski[2], Phil F. Battley[3], Zhijun Ma[4], Shufen Yang[1], Choon Beng How[1], Doug Watkins[5], Philip Round[6], Alex Yee[1], Vupasana Srinivasan[1], Clarice Teo[1], Robert Teo[1], Adrian Loo[1], Chee Chiew Leong[1], Kenneth Er[1]
1 – National Parks Board, Singapore, 718925, Singapore
2 – Alfred-Wegener-Institute Helmholtz Centre for Marine and Polar Research, Potsdam, Germany
3 – Wildlife & Ecology Group, Massey University, Palmerston North, 4442, New Zealand
4 – Ministry of Education Key Laboratory for Biodiversity Science and Ecological Engineering, Coastal Ecosystems Research Station of the Yangtze River Estuary, Institute of Biodiversity Science, School of Life Sciences, Fudan University, Shanghai, 200433, China
5 – Australasian Wader Studies Group, Carlton VIC, 3053, Australia
6 – Department of Biology, Mahidol University, Bangkok, 10400, Thailand
Correspondence and requests for materials should be addressed to D. L. (email: David_Li@nparks.gov.sg)

Supplementary Information:

Supplementary Table S1. Bird ringing recoveries and flag resightings of Common Redshanks in Southeast and East Asia between 1965 and 2019.

No.	Flagging & ringing site[1]	Date flagged or ringed	Observation or recapture site	Date seen or recaptured	Source[2]	Method[3]
1	Chongming Dongtan Reserve, Shanghai, China		Xiaoyangkou, Rudong, Jiangasu, China	31/5/2011	5	2
2	Chongming Dongtan Reserve, Shanghai, China		Yancheng Nature Reserve, China	17/6/2009	5	2
3	Chongming Dongtan Reserve, Shanghai, China		Chiang-Chun, Tainan County, Taiwan area	4/9/2005	1, 5, 8	2
4	Chongming Dongtan Reserve, Shanghai, China		Chi-Ku, Tainan County, Taiwan area	25/8/2007	1, 5, 8	2
5	Chongming Dongtan Reserve, Shanghai, China		Hsia-Pu, Ilan County, Taiwan area	10/4/2010	1, 5, 8	2
6	Chongming Dongtan Reserve, Shanghai, China		Chi-Ku, Tainan County, Taiwan area	16/10/2012	5, 8	2
7	Chongming Dongtan Reserve, Shanghai, China		Mei-Shan, Hsin-chu City, Taiwan area	13/1/2013	5, 8	2
8	Chongming Dongtan Reserve, Shanghai, China		Hsin-pao, Changhwa County, Taiwan area	21/11/2014	5, 8	2
9	Chongming Dongtan Reserve, Shanghai, China		Hsiang-Shan, Hsinchu City, Taiwan area	6/5/2015	5, 8	2
10	Chongming Dongtan Reserve, Shanghai, China		Lake Ci Fishponds, Kinmen, Taiwan area	8/4/2018	1, 5	2
11	Chongming Dongtan Reserve, Shanghai, China		Kamisuda, Inashiki-shi, Ibaraki-ken, Japan	17/8/2008	3, 5	2
12	Chongming Dongtan Reserve, Shanghai, China		Olango Island, the Philippines	23/2/2007	1, 5	2
13	Chongming Dongtan Reserve, Shanghai, China		Olango Island, the Philippines	8/12/2008	1, 5	2
14	Chongming Dongtan Reserve, Shanghai, China		Olango Island, the Philippines	14/1/2011	1, 5	2
15	Chongming Dongtan Reserve, Shanghai, China		Olango Island, the Philippines	5/2/2011	1, 5	2
16	Chongming Dongtan Reserve, Shanghai, China		Tawau, Sabah, Malaysia	10/2/2019	18	2
17	Hangzhou bay, Zhejiang, China		Tawau, Sabah, Malaysia	23/2/2019	18	2
18	Mai Po Nature Reserve, Hong Kong, China	5/5/1979	Taishan County, Guangdong, China	27/4/1980	11	1
19	Mai Po Nature Reserve, Hong Kong, China	10/8/1988	Chenghai, Guangdong, China	10/4/1992	11	1
20	Mai Po Nature Reserve, Hong Kong, China	9/8/1989	Xuan Thuy, River River Delta, Vietnam	14/10/1992	11	1
21	Mai Po Nature Reserve, Hong Kong, China	9/9/1991	Laizhou Bay, Shandong, China	May/Jun 1992	11	1
22	Mai Po Nature Reserve, Hong Kong, China	20/8/1988	Yang Jiao Gou Salt Farm, Chouguang, Shandong, China	21/5/1993	11	1
23	Mai Po Nature Reserve, Hong Kong, China	2/11/2011	Nanpu, Bohai Bay, Hebei, China	4/5/2016	1, 7	2
24	Mai Po Nature Reserve, Hong Kong, China		Kapar Power Station Ash Ponds, Malaysia	13/11/2016	1, 7	2
25	Mai Po Nature Reserve, Hong Kong, China		Dashbalbar, Dornod, Mongolia	20/5/2019	7	2
26	Mai Po Nature Reserve, Hong Kong, China	27/4/2013	Fish Pond Wonorejo, Surabaya, Indonesia	24/8/2013	1, 7	2
27	Taipei-Kaohsiung, Taiwan area		Yalu Jiang National Nature Reserve, China	18/3/2010	1	2
28	Taipei-Kaohsiung, Taiwan area		Nanpu, Bohai Bay, Hebei, China	30/4/2018	1	2
29	Han-pao, Changhwa County, Taiwan area		Uratabaru, Nagura, Ishigaki-shi, Okinawa-ken, Japan	28/9/2006	1	2
30	Han-pao, Changhwa County, Taiwan area	4/10/2005	Uratabaru, Nagura, Ishigaki-shi, Okinawa-ken, Japan	29/10/2006	3, 8	2
31	Han-pao, Changhwa County, Taiwan area	27/8/2007	JujiangSaltfarm, Shijing, Nanan, Fujian, China	24/8/2008	4, 6	1
32	Han-pao, Changhwa County, Taiwan area		Tung-Kang, Yalu River Estuary, Liaoning, China	18/3/2010	8	2
33	Changhwa County, Taiwan area		Xing zhuang river mouth, Lian Yun Gang, China	18/5/2014	8	2
34	Szu-Tsao, Tainan City, Taiwan area	31/3/1990	Hangzhou Bay, Zhejiang, China	12/5/1990	4, 6	1
35	Szu-Tsao, Tainan City, Taiwan area	21/4/1990	Hangzhou Bay, Zhejiang, China	30/12/1990	4, 6	1
36	Szu-Tsao, Tainan City, Taiwan area		Nagura-anbaru, Ishigaki-shi, Okinawa-ken, Japan	28/9/2006	3	2
37	Szu-Tsao, Tainan City, Taiwan area	31/10/1999	Szu-Tsao, Tainan City, Taiwan area	7/2/2012	8	2
38	Szu-Tsao, Tainan City, Taiwan area	13/9/2012	Sankaku-ike, Tomigusuku-shi, Okinawa-ken, Japan	17/8/2017	3, 8	1
39	Jambi, Sumatra, Indonesia		Khok Kham, Samut Sakhon, Thailand	12/8/2008	1	2

No.	Flagging & ringing site[1]	Date flagged or ringed	Observation or recapture site	Date seen or recaptured	Source[2]	Method[3]
40	Jambi, Sumatra, Indonesia		Pools between Lujuhe & Haibin Yuchang, Tianjin, China	28/7/2009	1	2
41	Jambi, Sumatra, Indonesia		Kalong, Samut Sakhorn province, Thailand	3/8/2010	1	2
42	Jambi, Sumatra, Indonesia		Khok Kham, Samut Sakhon province, Thailand	21/8/2012	9	2
43	Jambi, Sumatra, Indonesia		Hongyuan grassland, Sichuan, China	15/5/2015	12	2
44	Jambi, Sumatra, Indonesia		Salt Pond, Subei County, Gansu, China	9/6/2016	1	2
45	Jambi, Sumatra, Indonesia		Pasir Debu prawn-ponds, near Penaga, Pinang, Malaysia	11/8/2010	1	2
46	Shunkunitai, Nemuro-shi, Hokkaido, Japan		Lin-Pien, Pingtung County, Taiwan area	23/4/2000	3, 8	2
47	Shunkunitai, Nemuro-shi, Hokkaido, Japan		Onaga Tidal Flat, Naha, Okinawa, Japan	22/4/2009	3, 1	2
48	Lake Komuke, Monbetsu-shi, Hokkaido, Japan	12/9/2011	Sohara-cho, Matsusaka-shi, Mie-ken, Japan	8/10/2011	3	2
49	Torinoumi, Watari-cho, Miyagi-ken, Japan	2/9/2013	Ushigome, Kisarazu-shi, Chiba-ken, Japan	22/12/2013	3	2
50	Torinoumi, Watari-cho, Miyagi-ken, Japan	2/9/2013	Ushigome, Kisarazu-shi, Chiba-ken, Japan	28/10/2014	3	2
51	Hiyagon tidal-flat, Okinawa, Okinawa, Japan	24/1/2008	Tai-Ping estuary, Taitung County, Taiwan area	22/9/2011	3	1
52	Hiyagon tidal-flat, Okinawa, Okinawa, Japan	25/2/2014	Yatabe, Kamisu-shi, Ibaraki-ken, Japan	8/4/2014	3	2
53	Hiyagon tidal-flat, Okinawa, Okinawa, Japan	26/2/2014	Hengsha Island, Shanghai, China	7/9/2014	3	2
54	Hiyagon tidal-flat, Okinawa, Okinawa, Japan	6/5/2013	Yalujiang National Nature Reserve, China	26/6/2016	1, 3, 8	2
55	Hiyagon tidal-flat, Okinawa, Okinawa, Japan	4/3/2012	Dapeng Bay, Pingtung County, Taiwan	13/9/2014	8	2
56	Hiyagon tidal-flat, Okinawa, Okinawa, Japan	4/3/2012	Dapeng Bay, Pingtung County, Taiwan	22/10/2016	3, 8	2
57	Hiyagon tidal-flat, Okinawa, Japan	4/3/2012	Dapeng Bay, Pingtung County, Taiwan	30/8/2018	3, 8	2
58	Kuala Selangor, Malaysia*	27/11/1987	Xining, Qinghai, China	Oct 1990	4, 6, 10	1
59	Tanjung Kerang, Selangor, Malaysia	21/4/1989	Laem Phak Bia, Thailand	10/9/2006	9, 23	1
60	Kuala Gula, Perak, Malaysia	22/10/2017	Near Hongyuan airport, Sichuan, China	Jul 2018	10, 15	1
61	Oriental Phil Alangilanon Manjuyuod, Philippine	15/10/1965	Limahong Channel, Pangasinan, Luzon, Philippine	12/9/1966	20	1
62	Oriental Phil Alangilanon Manjuyuod, Philippine	5/11/1967	Vyazemsky district, prominent, Khabarovsk, Russia	28/4/1968	2, 20	1
63	Iwahig, Palawan, Philippine	26/8/1967	Lingayen, Pangasinan, Luzon, Philippine	8/8/1969	20	1
64	Odoptu Bay, Sakhalin, Russia		Oki beach, Amihoshi-ku, Hyogo-ken	21/9/2011	2, 3	2
65	Serangoon, Singapore	13/9/1983	Pattani Bay, Thailand	22/7/1985	22, 23	1
66	Sungei Buloh Wetland Reserve, Singapore		Xiao Bei Hu Wetlands, Qinghai Lake, China	7/6/2012	17	2
67	Sungei Buloh Wetland Reserve, Singapore		Xiao Bei Hu Wetlands, Qinghai Lake, China	14/8/2018	24	2
68	Sungei Buloh Wetland Reserve, Singapore	7/12/2012	Lhalu Wetland, Lasha, Xizang, China	10/6/2013	13, 16	2
69	Sungei Buloh Wetland Reserve, Singapore		Mianyang, Sichuan, China	12/4/2015	15	2
70	Sungei Buloh Wetland Reserve, Singapore	18/11/2013	Khok Kham, Samut Sakhon, Thailand	31/7/2018	14	2
71	Sungei Buloh Wetland Reserve, Singapore		Khok Kham, Samut Sakhon, Thailand	17/8/2008	1	2
72	Sungei Buloh Wetland Reserve, Singapore	22/10/2013	Outside Khao Sam Roi Yot National Park, Thailand	1/9/2018	19	2
73	Sungei Buloh Wetland Reserve, Singapore	16/10/2017	Bang Pu, Samut Prakan, Thailand	2/8/2018	9	2
74	Sungei Buloh Wetland Reserve, Singapore	16/10/2017	Bang Pu, Samut Prakan, Thailand	26/9/2018	9	2
75	Sungei Buloh Wetland Reserve, Singapore	16/10/2017	Teluk Air Tawar, Butterworth, Malaysia	2/5/2019	21	2
76	Laem Phak Bia, Phetchaburi, Thailand	27/8/2006	Jambi, Sumatra, Indonesia	28/4/2008	9	1
77	Inner Gulf of Thailand		Tanjung Tokong, Penang, Malaysia	7/10/2006	1, 23	2
78	Inner Gulf of Thailand		Tanjung Tokong, Penang, Malaysia	5/3/2007	1	2
79	Inner Gulf of Thailand		Pulau Burung, Nibong Tebal, Penang, Malaysia	29/8/2007	1	2
80	Inner Gulf of Thailand		Parit Penyengat Laut, Muar, Johor, Malaysia	22/12/2007	1	2
81	Inner Gulf of Thailand		Parit Penyengat Laut, Muar, Johor, Malaysia	4/10/2008	1	2
82	Inner Gulf of Thailand		Kg. Sri Menanti, Johor, Malaysia	10/9/2010	9	2
83	Inner Gulf of Thailand		Kapar Power Station Ash Ponds, Malaysia	10/9/2010	1	2
84	Inner Gulf of Thailand		Sungei Buloh Wetland Reserve, Singapore	12/10/2006	1, 23	2
85	Inner Gulf of Thailand		Sungei Buloh Wetland Reserve, Singapore	27/7/2009	1	2

Notes:

All dates are indicated by day/month/year.

Flagging & ringing site[1]: *- Zhang & Yang (1997) reported the bird ringed at Kuching, East Malaysia, it was later confirmed to have come from Kuala Selangor, West Malaysia (Ahmad Khusaini Bin Mohd Kharip Shah, Department of Wildlife and National Parks (Perhilitan) Peninsular Malaysia pers. comm.).

Source[2]: 1-Australasian Wader Studies Group; 2-Bird Ringing Centre of Russia, IEE RAS; 3-Yamashina Institute for Ornithology; 4-China National Bird Banding Center; 5-Shanghai Chongming Dongtan Nature Reserve; 6- Zhang F.Y. & Yang R.L. *China Bird Migration Research* (in Chinese). (Forestry Press, Beijing, 1997); 7-Hong Kong Waterbirds Ringing Group; 8-Taiwan Wader Study Group; 9-Philip Round pers. comm. (Thailand); 10-Wildlife Department Malaysia; 11- Carey, G.J., M.L. Chalmers, D.A. Diskin, P.R. Kennerley, P.J. Leader, M.R. Leve, R.W. Lewthwaite, D.S. Melville, M. Turnbull, & L. Young. 2001. *The Avifauna of Hong Kong*. (Hong Kong Bird Watching Society, 2001); 12-Standen, R. & Londo, I. Sumatran-flagged Common Redshank seen on the breeding grounds. *Tattler* 37, 7–8 (2015); 13- Lu, X. *The birds of Qinghai-Tibet Plateau of China*. (In Chinese). (Hunan Science and Technology Press, Changsha, 2018); 14-Bung Boraphet Wildlife Research Station; 15-Hao Liu pers. comm.; 16-Hongfen Cao pers. comm.; 17- Chia, A. A 'Ringing' Endorsement for Singapore Migrant's Flight of Wonder. *Nature Watch* 21, 17 (2013); 18-Stanley Shao pers. comm; 19-Charles Currin pers. comm.; 20- McClure, H. E. *Migration and survival of the birds of Asia*. (US Army Medical Component, SEATO Medical Project, Bangkok, 1974); 21-Nur Munira pers. comm.; 22-Watkins, D. Sungei Buloh Project, Singapore. *Shorebird Banding Workshop - Oct-Nov.1990.* (Report to Asian Wetland Bureau 1990); 23-Round, P.D. & D. Gardner. *The birds of the Bangkok area*. (White Lotus, Bangkok 2008); 24- WenXiao Dong pers. comm.

Method[3]: 1- Ringing recovery; 2- Flag observation.

Supplementary Table S2. Individual migratory movements of adult Common Redshanks from and to Sungei Buloh Wetland Reserve, Singapore, determined using light-level geolocation (Geo) and satellite tracking (PTT).

Tracker No.	Tag type	Northward migration					Days at breeding grounds	Southward migration				
		Date depart non-breeding grounds	No. of key stopover sites / days used[1]	Date arrive breeding grounds	Days travelling	Migration distance N (km)[2]		Date depart breeding grounds	No. of key stopover sites / days used[1]	Date arrive non-breeding grounds	Days travelling	Migration distance S (km)[2]
P332[3]	Geo	9/5/2015	0/0	13/5/2015	4	4054	103	24/8/2015	1/3	Nil	Nil	
P359/ 36139[3]	Geo	13/3/2015	2/62	22/5/2015	70	4341	98	28/8/2015	0/0	2/9/2015	5	3964
P359/ 36139[3]	PTT	8/3/2018	2/69	19/5/2018	72	3897	103	30/8/2018	Nil	Nil[8]	Nil	Nil
36134[3]	PTT	6/5/2018	2/10	21/5/2018	15	3735	99	28/8/2018	1/21	23/9/2018	26	3891
36134[3]	PTT	1/5/2019	1/9	17/5/2019	16	3829	105	30/8/2019	2/14	25/9/2019	26	3972
P385[4]	Geo	4/4/2015	1/7	19/4/2016	15	4999	74	2/7/2016	2/11	26/7/2015	24	6571
P809[4]	Geo	1/4/2015	1/11	17/4/2015	16	4525	82	8/7/2015	1/9	23/7/2015	15	4010
36135[4]	PTT	16/4/2018	1/25	18/5/2018	32	4658[7]	95	21/8/2018	4/31	2/10/2018	42	4171
P352	Geo	8/5/2015	1/3	21/5/2015	13	4994	105	3/9/2015	0/0	12/9/2015	9	4383
P812	Geo	8/5/2015	0/0	13/5/2015[5]	5	4116	Nil	Nil	Nil	Nil	Nil	Nil
P817	Geo	13/4/2015	2/29	Nil[6]	38+	Nil	Nil	Nil	Nil	Nil	Nil	Nil
P820	Geo	13/4/2015	0/0	16/4/2015	3	4920	104	29/7/2015	0/0	2/8/2015	4	5635
P820	Geo	8/4/2016	0/0	11/4/2016	3	4617	105	25/7/2016	0/0	29/7/2016	4	5009

Notes:

All dates are indicated by day/month/year in SGT (UTC+8).

1 – For the list of stopover sites see Table S4; for criteria of stopover sites see methods.

2 – Migration distance calculated as summed great circle distances between consecutive locations between non-breeding and breeding grounds for both geolocator tagged birds (median points of modelled tracks) and PTT-tagged birds (high quality locations).

3 – Birds that took a direct Himalayan crossing during both northward and southward migration.

4 – Birds that took a direct Himalayan crossing during southward migration only.

5 – Tracker stopped on 2 August at breeding grounds.

6 – Tracker stopped on 21 May during northward migration.

7 – Distance to post-breeding grounds.

8 – Tracker stopped on 2 Sep during southward migration.

Supplementary Table S3. Individual migratory movements of adult Whimbrels from and to Singapore using satellite tracking.

Tracker No.	Northward migration					Days at breeding grounds	Southward migration				
	Date depart non-breeding grounds	No. of key stopover sites / days used[1]	Date arrive breeding grounds	Days travelling	Migration distance N (km)[2]		Date depart breeding grounds	No. of key stopover sites / days used[1]	Date arrive non-breeding grounds	Days travelling	Migration distance S (km)[2]
36133	23/4/2019	1/22	27/5/2019	34	8,353	63	29/7/2019	2/50	24/9/2019	57	8,317
168746	30/4/2018	2/16	7/6/2018	38	9,019	61	7/8/2018	2/42	30/9/2018	54	8,398
168746	28/4/2019	3/21	1/6/2019	34	9,041	63	3/8/2019	3/39	24/9/2019	52	8,530
168748	3/5/2018[4]	3/28	Nil	Nil	Nil	Nil	Nil	Nil	Nil	Nil	Nil
168749	21/4/2018	2/19	24/5/2018	33	8,557	67	30/7/2018	3/49	2/10/2018	64	8,764
168749	20/4/2019	2/24	24/5/2019	34	8,266	66	29/7/2019	3/33	15/9/2019	48	8,111
168750[3]	26/4/2018	2/31	15/6/2018	50	8,039	61	15/8/2018	4/100	14/12/2018	121	8,619
168750[3]	25/4/2019	1/14	4/6/2019	40	8,114	83	26/8/2019	2/28	5/10/2019	40	8,556

Notes:

All dates are indicated by day/month/year in SGT(UTC+8).

1 – For the criteria of stopover site see methods, for a full list of stopover sites see Table S6.

2 – Migration distance calculated as summed great circle distances between consecutive locations between non-breeding and breeding ground from high quality locations.

3 – Birds that took a direct Himalayan crossing during both northward and southward migration.

4 – Tracker stopped on 11/6/2018 during northward migration.

Supplementary Table S4. List of stopover sites and breeding grounds of adult Common Redshanks from and to Sungei Buloh Wetland Reserve, Singapore, determined using light-level geolocation and satellite tracking.

Tracker No.	Locations	Coordinates[1]	Date (no. of days)	Stopover sites or breeding grounds[2]
P332	Golmud, Qinghai, China	35.79°N (35.71°N-35.89°N), 93.37°E (93.30°E-93.42°E)	13/5–24/8/2015 (103)	Breeding grounds
P332	Inner Gulf of Thailand	14.04°N (10.48°N -18.13°N), 100.97°E (100.11°E -101.80°E)	27–30/8/2015 (3)	Southward migration
P352	Border of Myanmar and China	29.31°N (28.07°N -30.75°N), 99.03°E (98.24°E -99.80°E)	15–18/5/2015 (3)	Northward migration
P352	Ulan County, Qinghai, China	36.68°N (36.56°N -36.81°N), 98.96°E (98.88°E -99.04°E)	21/5–3/9/2015 (105)	Breeding grounds
P359	Southern Malaysia/Central Sumatra	1.32°N (-1.89°N -4.80°N), 102.45°E (102.01°E -102.93°E)	14/3–26/4/2015 (42)	Northward migration
P359	Likely Gulf of Mottama, Myanmar	16.18°N (14.56°N -18.06°N), 97.37°E (96.65°E -98.07°E)	28/4–18/5/2015 (20)	Northward migration
P359	Nagqu, Xizang/Golmud, Qinghai border, China	33.42°N (33.01°N -33.84°N), 90.68°E (90.38°E -90.97°E)	20/5–29/8/2015 (98)	Breeding grounds
P385	Ngawa, Sichuan, China	32.96°N (31.62°N -34.47°N), 102.87°E (102.45°E -103.32°E)	7–14/4/2015 (7)	Northward migration
P385	Haixi, Qinghai, China	37.86°N (37.72°N -38.02°N), 97.28°E (97.15°E -97.41°E)	19/4–2/7/2015 (74)	Breeding grounds
P385	Myanmar coast	14.97°N (13.47°N -16.98°N), 95.60°E (94.75°E -96.49°E)	8–11/7/2015 (3)	Southward migration
P385	Inner Gulf of Thailand	12.63°N (11.81°N -13.56°N), 100.48°E (100.08°E -100.90°E)	15–23/7/2015 (8)	Southward migration
P809	Linxia, Gansu, China	35.23°N (34.32°N -36.34°N), 103.44°E (103.08°E -103.80°E)	3–14/4/2015 (11)	Northward migration
P809	Golog, Qinghai, China	35.29°N (35.14°N -35.45°N), 98.05°E (97.95°E -98.16°E)	17/4–8/7/2015 (82)	Breeding grounds
P809	Inner Gulf of Thailand	13.07°N (12.44°N -13.85°N), 100.87°E (100.52°E -101.20°E)	12–21/7/2015 (9)	Southward migration
P812	Yushu, Qinghai, China	34.35°N (34.23°N -34.49°N), 93.36°E (93.26°E -93.48°E)	12/5–2/8/2015 (82, tracker stopped)	Breeding grounds
P817	Southern Myanmar	15.59°N (14.61°N -16.74°N), 98.21°E (97.87°E -98.53°E)	15/4–7/5/2015 (22)	Northward migration
P817	Garze, Sichuan, China	30.35°N (29.52°N -31.53°N), 101.37°E (100.75°E -101.93°E)	10–17/5/2015 (7, tracker stopped)	Northward migration
P820	Possibly Inner Mongolia/ Gansu Boarder, China	39.03°N (38.61°N -39.66°N), 101.56°E (101.27°E -101.84°E)	16/4–25/7/2015 (9)	Pre-breeding grounds
P820	Haixi, Qinghai, China	36.61°N (36.55°N -36.70°N), 97.65°E (97.60°E -97.71°E)	28/4–10/7/2015 (73)	Breeding grounds
P820	Gansu and Qinghai Border, China	38.06°N (37.83°N -38.39°N), 101.39°E (101.13°E -101.62°E)	13–29/7/2015 (16)	Post-breeding grounds
P820	Haibei, Qinghai, China	37.29°N (36.19°N -38.17°N), 100.68°E (100.20°E -101.21°E)	11–16/4/2016 (5)	Pre-breeding grounds
P820	Gansu/Qinghai border, China	39.01°N (38.92°N -39.11°N), 99.49°E (99.40°E -99.57°E)	18/4–25/7/2016 (98)	Breeding grounds
36134	Hmau Bhi Kyun coast, Myanmar	16.19°N, 95.89°E	8–15/5/2018 (7)	Northward migration
36134	Zhegu Co, Shannan, Xizang, China	28.69°N, 91.66°E	18–20/5/2018 (3)	Northward migration
36134	Southeast end of Siling Cuo, Xizang, China	31.54°N, 89.17°E	21/5–28/8/2018 (99)	Breeding grounds
36134	Gulf of Mottama, Myanmar	16.88°N, 96.84°E	31/8 –21/9/2018 (21)	Southward migration
36134	Gulf of Mottama, Myanmar	17.16°N, 97.02°E	3–12/5/2019 (9)	Northward migration
36134	Southeast end of Siling Cuo, Xizang, China	31.54°N, 89.17°E	17/5–30/8/2019 (105)	Breeding grounds
36134	Gulf of Mottama, Myanmar	16.99°N, 96.80°E	4–15/9/2019 (11)	Southward migration
36134	northern Selangor coast, Malaysia	3.54°N, 101.07°E	18–20/9/2019 (3)	Southward migration
36135	Laem Sing District, Chanthaburi province, Inner Gulf of Thailand	12.39°N, 102.16°E	19/4–14/5/2018 (25)	Northward migration
36135	Hongyuan, Aba, Sichuan Province, China	32.46°N, 102.40°E	18/5–15/7/2018 (58)	Breeding grounds
36135	Gangnagema Cuo, Madoi, Golog, Qinghai Province, China	34.33°N, 98.62°E	17/7–21/8/2018 (35)	Post-breeding grounds
36135	Gulf of Mottama, Myanmar	16.97°N, 96.83°E	25/8–4 /9/2018 (10)	Southward migration
36135	Prachuap Khiri Khan, Thailand	11.83°N, 99.80°E	7–15 /9/2018 (8)	Southward migration
36135	Thale Noi National Park, Thailand	7.69°N, 100.35°E	16–23 /9/2018 (7)	Southward migration
36135	Batu Pahat coast, Johor, Malaysia	1.80°N, 102.89°E	25 /9–1/10/ 2018 (6)	Southward migration
36139	Kelang Islands, Selangor, Malaysia	2.95ºN, 101.19ºE	9/3–28/4/2018 (50)	Northward migration
36139	Gulf of Mottama, Myanmar	17.16ºN, 97.03ºE	29/4–18/5/2018 (19)	Northward migration
36139	Zagya Zangbo River in Nagqu, Xizang, China	32.19ºN, 89.99 ºE	19/5–24/7/2018 (56 excluding 21–31 May)	Breeding grounds
36139	Jiang Jia Cuo, Nagqu, Xizang, China	31.97ºN, 89.84 ºE	21–31/5/2018 (10)	Breeding grounds
36139	Namuka Cuo, Nagqu, Xizang, China	31.88ºN, 89.75 ºE	22–30/8/2018 (8)	Post-breeding grounds

Notes:

All dates are indicated by day/month/year.

1 – Coordinates for geolocator tagged birds shown as median point and 95% credibility interval.

2 – For the criteria of stopover site see methods.

Supplementary Table S5. Mean elevations of trans-Himalayan and eastern migrations of Common Redshanks and Whimbrels.

		Trans–Himalayan (m asl)		Eastern (m asl)			
Species	Migration	Mean±SD	N	Mean±SD	N	Z	P
Common Redshank	North	871±321	4	968±358	7	0.4665	0.6847
	South	1402±579	4	1386±494	4	-0.0581	0.9673
	Overall	1136±518	8	1140±452	12	0.0204	0.9851
Whimbrel	North	999±120	2	330±45	3	-1.9664	0.0998
	South	956±124	2	304±45	3	-1.9511	0.1022
	Overall	977±102	4	316±56	6	-2.9344	0.0048

Note:
For Redshanks, Eastern refers to route circumventing the Himalayan crossing. For Whimbrel, Eastern refers to coastal East Asia.

Supplementary Table S6. List of stopover sites and breeding grounds of adult Whimbrels from and to Singapore determined using satellite tracking.

Tracker No.	Locations	Coordinates	Date (no. of)	Stopover sites or breeding grounds
36133	Haixing, Cangzhou, Hebei, China	38.17°N, 117.75°E	30/4–22/5/2019 (22)	Northward migration
36133	Olenyoksky District, Sakha Republic, Russia	69.84°N, 110.50°E	27/5–29/7/2019 (63)	Breeding grounds
36133	Yangcheng Reservior, Cangzhou, Hebei, China	38.17°N, 117.75°E	31/7–16/9/2019 (47)	Southward migration
36133	Pahang and Johor coast, Malaysia	3.98°N, 103.44°E - 2.65°N, 103.70°E	20–22/9/2019 (3)	Southward migration
168746	Jiuduansha, Shanghai, China	31.22°N, 122.03°E	14–24/5/2018 (10)	Northward migration
168746	Sandao Reservoir, Songyuan City, Jilin, China	45.38°N, 125.56°E	28/5–3/6/2018 (6)	Northward migration
168746	Taymyrsky Dolgano-Nenetsky District, Krasnoyarsk Krai, Russia	70.26°N, 106.97°E	7/6–7/8/2018 (61)	Breeding grounds
168746	Yaitai, Shangdong, China	37.43°N, 121.55°E	11/8–5/9/2018 (25)	Southward migration
168746	Vinh Van Phong, Khanh Hoa, Vietnam	12.61°N, 109.20°E	10–27/9/2018 (17)	Southward migration
168746	Aiwen Bay, Zhejiang, China	28.28°N, 121.58°E	1–8/5/2019 (7)	Northward migration
168746	Laiyang, Shandong, China	36.64°N, 120.69°E	13–23/5/2019 (10)	Northward migration
168746	Zhaoyuan, Heilongjiang, China	45.43°N, 125.38°E	25–29/5/2019 (4)	Northward migration
168746	Taymyrsky Dolgano-Nenetsky District, Krasnoyarsk Krai, Russia	70.26°N, 106.98°E	1/6–3/8/2019 (63)	Breeding grounds
168746	Huludao, Liaoning, China	39.99ºN, 119.88°E	7–19/8/2019 (12)	Southward migration
168746	Laishan, Yantai, Shandong, China	37.44°N, 121.55°E	22/8–6/9/2019 (15)	Southward migration
168746	Van Hung, Van Niah, Khanh Hoa, Vietnam	12.61ºN, 109.20°E	10–22/9/2019 (12)	Southward migration
168748	Yangtze River at Yichang City, Hubei, China	30.41°N, 111.46°E	5–8/5/2018 (3)	Northward migration
168748	Binhai, Tianjin coast, China	38.89°N, 117.54°E	15–20/5/2018 (5)	Northward migration
168748	Mogochinskiy Rayon, Zabaykalsky Krai, Russia	54.27°N, 121.02°E	22/5–11/6/2018 (20)	Northward migration
168749	Quanzhou, Fujian, China	24.67°N, 118.44°E	25–29/4/2018 (4)	Northward migration
168749	Taeryong River mouth, DPR Korea	39.52ºN, 125.42°E	4–19/5/2018 (15)	Northward migration
168749	Verkhoyansky District, Sakha Republic, Russia	67.65°N, 131.68°E	24/5–30/7/2018 (67)	Breeding grounds
168749	Baicheng, Jilin, China	46.03°N, 123.28°E	2–5/8/2018 (3)	Southward migration
168749	Hongzi Lake, Songyuan, Jilin, China	45.20ºN, 124.03°E	7–29/8/2018 (22)	Southward migration
168749	Changpi Port, Hainan Island, China	19.46ºN, 110.79°E	5–29/9/2018 (24)	Southward migration
168749	Hwanghae, DPRK	37.84°N, 126.22°E	26–28/4/2019 (3)	Northward migration
168749	Taeryong River mouth, DPRK	39.52°N, 125.42°E	30/4–21/5/2019 (21)	Northward migration
168749	Verkhoyansky District, Sakha Republic, Russia	67.65°N, 131.68°E	24/5–29/7/2019 (66)	Breeding grounds
168749	Hongzi Lake, Jilin, China	45.18°N, 124.07°E	1–22/8/2019 (21)	Southward migration
168749	My Xuan, Ba Ria - Vung Tau, Southern Vietnam	10.62°N, 107.02°E	27–29/8/2019 (3)	Southward migration
168749	An Ngai, Ba Ria - Vung Tau, Southern Vietnam	10.43°N, 107.21°E	3–12/9/2019 (9)	Southward migration
168750	Gulf of Mottama, Myanmar	17.15°N, 96.81°E	30/4–16/5/2018 (16)	Northward migration
168750	Mizhi Lake, Shuanghu, Nagqu, Xizang, China	36.26°N, 88.89°E	20/5–4/6/2018 (15)	Northward migration
168750	Tazovsky District, Yamalo-Nenets Autonomous Okrug, Russia	67.65°N, 79.90°E	15–21/6/2018 (6)	Breeding grounds
168750	Turukhansky District, Krasnoyarsk Krai, Russia	67.91°N, 82.50°E	23/6–15/8/2018 (53)	Breeding grounds
168750	Ozero Bychye, Altai Krai, Russia	51.83°N, 80.22°E	18–31/8/2018 (13)	Southward migration
168750	Bosten Lake, Xinjiang, China	42.00°N, 86.84°E	2–20/9/2018 (18)	Southward migration
168750	Bhitarkanika National Park, Odisha, India	20.68°N, 86.86°E	24/9–26/10/2018 (32)	Southward migration
168750	Krabi Estuary, Thailand	8.02°N, 98.93°E	31/10–7/12/2018 (37)	Southward migration
168750	Gulf of Mottama, Myanmar	17.20°N, 96.84°E	28/4–12/5/2019 (14)	Northward migration
168750	Turukhansky District, Krasnoyarsk Krai, Russia	67.91°N, 82.52°E	4/6–26/8/2019 (83)	Breeding grounds
168750	Lodipur Utrauwa, Uttar Pradesh, India	26.15°N, 81.04°E	31/8—6/9/2019 (6)	Southward migration
168750	Bhitarkanika National Park, Odisha, India	20.67ºN, 86.91°E	10/9–2/10/2019 (22)	Southward migration

Note: For the criteria of stopover site see methods.

Supplementary Table S7. Bird ringing recoveries and flag re-sightings of shorebirds from Southeast Asia and Australia possibly taking a westerly migration route or Trans-Himalayan crossing

No.	Species	Flagging & ringing site	Date flagged or ringed	Observation or recapture site	Date seen	Source[1]	Method[2]
1	Curlew Sandpiper	Werribee Sewage Farm, Melbourne, Victoria, Australia	Nov 1976	Point Calimere, India	Aug 1980	1	1
2	Curlew Sandpiper	Victoria, Australia		Chilika Lake, India	Not available	1	2
3	Curlew Sandpiper	Western shore of Alakoul Lake, Kazakhstan	19/7/1991	Kuala Kurau, Perak, Malaysia	6/2/1992	2	1
4	Curlew Sandpiper	Kuala Selangor, Malaysia	Jan 1978	Kazakhstan (76.05E)	Aug 1978	2, 3	1
5	Curlew Sandpiper	Northwest Australia		Bundala National Park, Sri Lanka	20/8/2005	6	2
6	Sanderling	Eighty Mile Beach, Australia	2/3/2013	Modhava Coast, Mandvi, Gujarat, India	21/11/2013	1, 4	2
7	Terek Sandpiper	Orange flag, North-west Australia		Kan Maw island, Myanmar	25/11/2016	5	2
8	Lesser Sand Plover	Sungei Buloh Wetland Reserve, Singapore		Gulf of Mottama, Myanmar	12/3/2013	7	2
9	Lesser Sand Plover	Ko Libong, Thailand	26/3/2009	Gulf of Mottama, Myanmar	Dec 2011	8	1
10	Lesser Sand Plover	Ko Libong, Thailand	29/3/2009	Gulf of Mottama, Myanmar	21/5/2013	8	1
11	Lesser Sand Plover	Kapar Power Station, Malaysia	8/12/2007	Gulf of Mottama, Myanmar	Not available	8	1
12	Lesser Sand Plover	Inner Gulf of Thailand		Tianquan River, Tianquan County, Sichuan, China	2/5/2019	9	2
13	Asian Dowitcher	Alabana Island of Chilika Lake, India	Mar 2016	Samut Sakhon, Thailand	15/8/2018	8	2

Notes:

All dates are indicated by day/month/year.

[1]Source: 1- Balachandran, S., Katti, T. & Manakadan, R. *Indian Bird Migration Atlas*. (Bombay Natural History Society & Oxford University Press, New Delhi, 2018); 2- Yatim, S.H. Short Notes on Band Recovery of Waders in 1991/1992. *Journal of Wildlife and Parks* 11, 58 – 59 (1991); 3- Wells, D. R. The *Birds of Thai-Malay Peninsula*, Volume 1: Non Passerines. (Academic Press, 1999); 4- Tiwari, J.K. An Australian Ringed Bird seen in Kutch, India. *Tattler* 31, 19 (2013); 5- Zöckler, C., Moses, S. & Lwin, S.T. The importance of the Myeik mangroves and mudflats, Tanintharyi, Myanmar for migratory waders and other waterbirds. *Wader Study* 126, 129-141 (2019); 6- Bellio, M. & Kaluthota, C. Australian Curlew Sandpiper on passage through Sri Lanka, *Wader Study Group Bull*. 110, 66 (2006); 7- Hiroshi Tomida pers. comm; 8 - Bung Boraphet Wildlife Research Station, Department of National Parks, Wildlife and Plant Conservation, Thailand; 9 – Pinjia Jue pers. comm.

[2]Method: 1- Ringing recovery; 2- Colour flag observation.

Supplementary Table S8. Summary of Common Redshanks and Whimbrels equipped with geolocators and PTT satellite transmitters in Singapore, 2015–2019.

	Geolocator tagging of Common Redshanks at Sungei Buloh Wetland Reserve (2015-2016)	Satellite tagging of Common Redshanks at Sungei Buloh Wetland Reserve (2018-2019)	Satellite tagging of Whimbrels at Sungei Buloh Wetland Reserve (2018-2019)	Satellite tagging of Whimbrels at Chek Jawa, Pulau Ubin (2018-2019)
No. of birds tagged	99	4	5	6
No. of tags retrieved	10	n/a	n/a	n/a
No. of birds with no data /remained at non-breeding ground without migration data collected[1]	1	1	0	3
No. of first year/ sub-adult birds carrying out partial migration (did not migrate to breeding grounds)	1	0	2	1
No. of adult birds with full or partial migration data available	8[2]	3[2]	3	2[2]

Notes:
n/a = not applicable
1 – Data from one geolocator was not able to be retrieved. One Redshank and two Whimbrels lost signals before migration and one sub-adult Whimbrel did not migrate.
2 – Three geolocator tagged Redshanks, one satellite tagged Redshank and one satellite tagged whimbrel did not record a full migration cycle.

Supplementary Figure S1. Individual migration routes of Common Redshanks based on light-level geolocation in 2015 and 2016 and satellite trackers in 2018 and 2019. One individual was tracked both by geolocator (P359) in 2015 and satellite tracker (36139) in 2018. Map Source: ETOPO1 1 Arc-Minute Global Relief Model dataset provided by NOAA https://www.ngdc.noaa.gov/mgg/global/global.html. Species distribution map provided by BirdLife International.

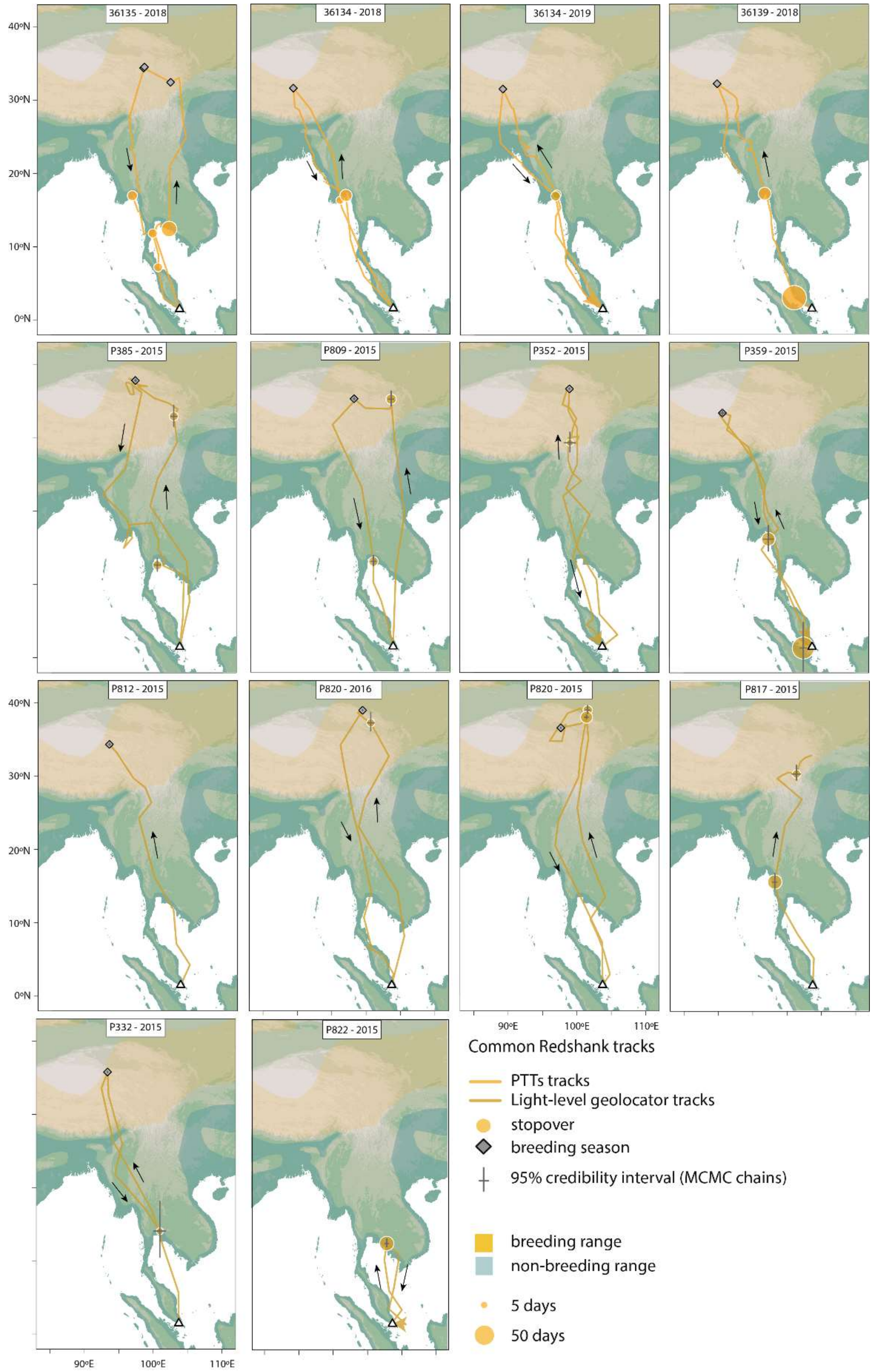

Supplementary Figure S2. Individual migration routes of Whimbrels based on satellite trackers in 2018 and 2019. Map Source: ETOPO1 1 Arc-Minute Global Relief Model dataset provided by NOAA https://www.ngdc.noaa.gov/mgg/global/global.html. Species distribution map provided by BirdLife International.

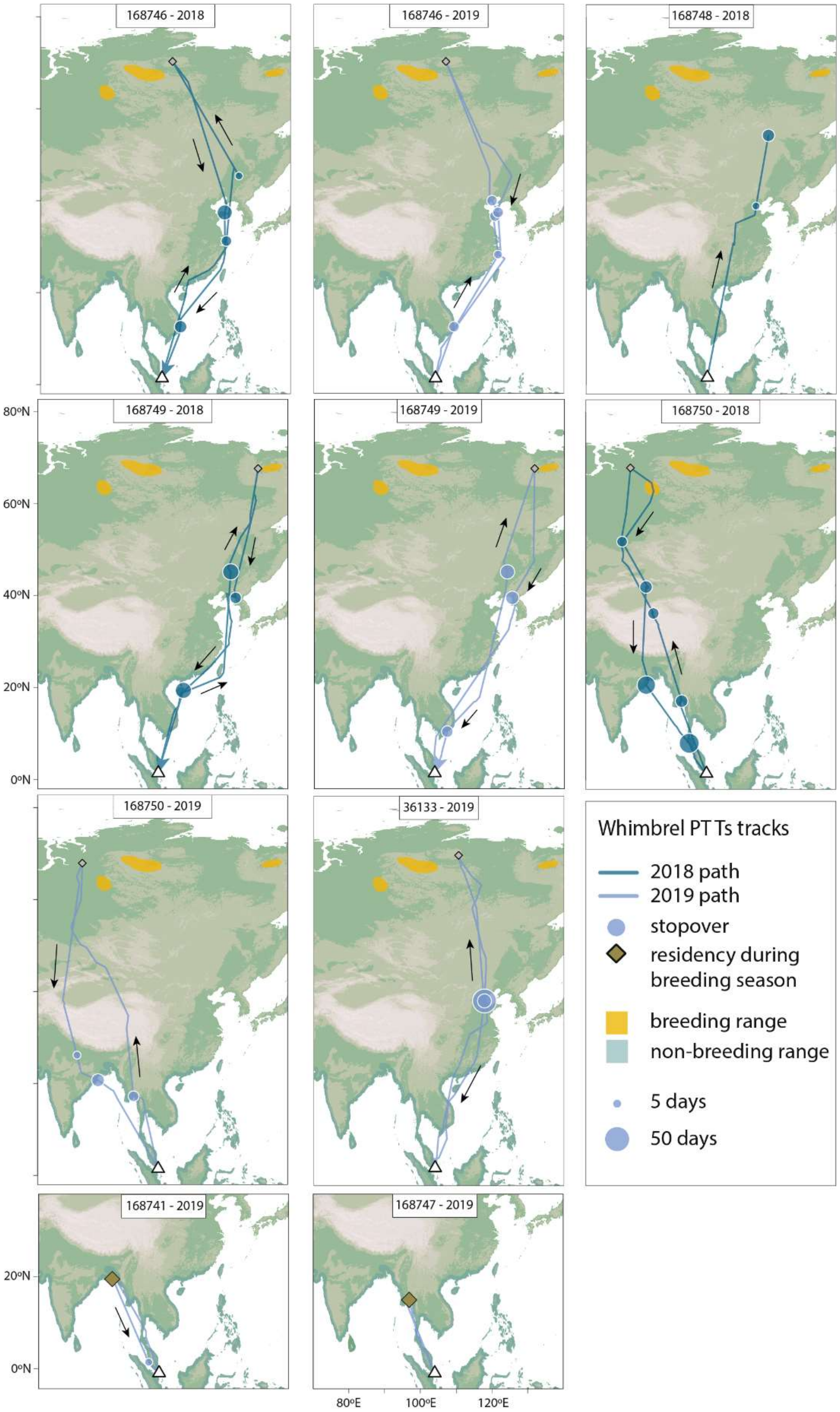

Supplementary Figure S3. Overall ground speeds recorded for Common Redshanks and Whimbrels by satellite tracking (PTT).

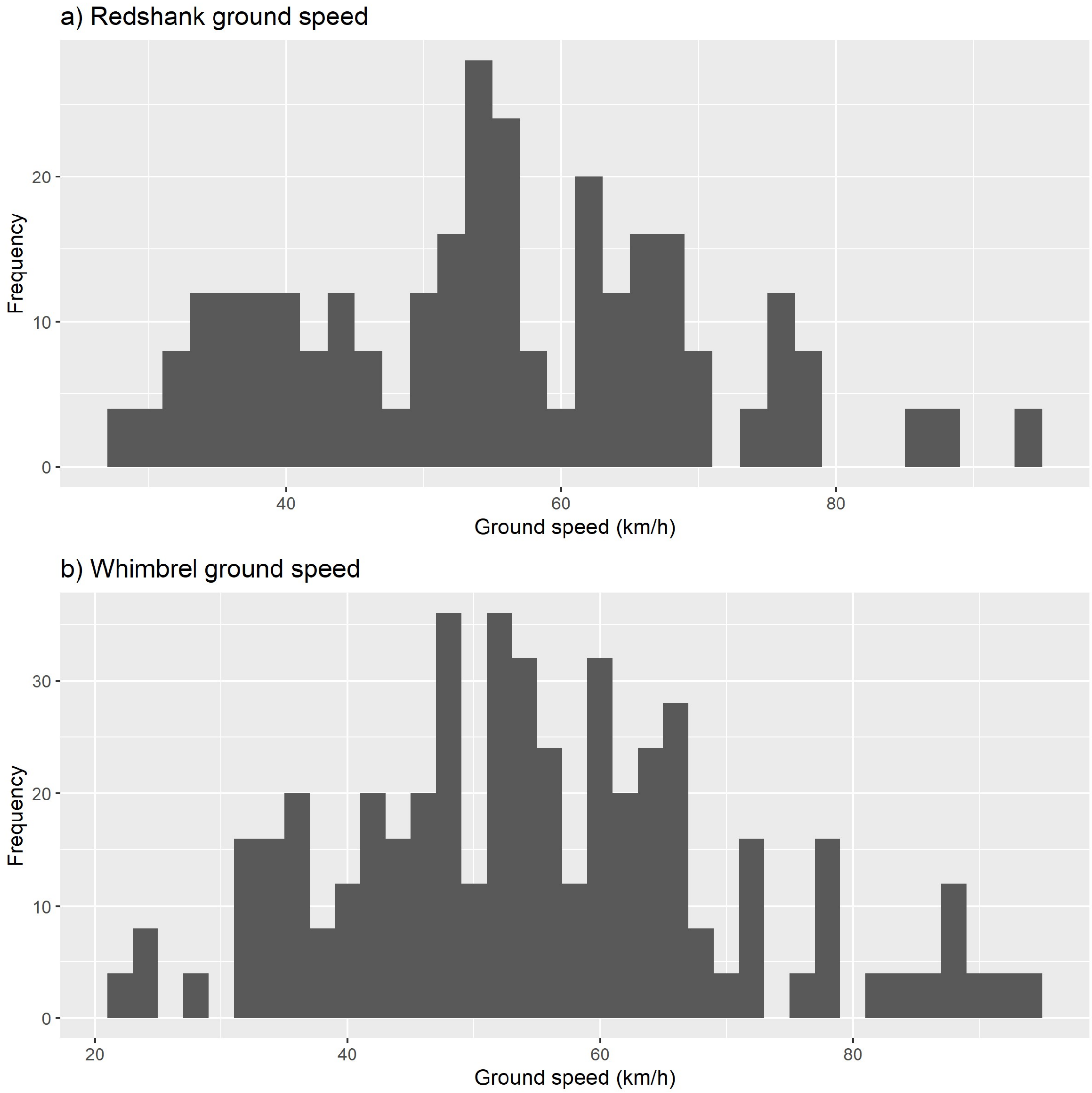

Supplement: Supplementary file 1 — Supplementary Information. [file 41598_2020_77897_MOESM1_ESM.pdf]
